# Cyclical endometrial repair and regeneration: Molecular mechanisms, diseases, and therapeutic interventions

**DOI:** 10.1002/mco2.425

**Published:** 2023-12-01

**Authors:** Xulin Hu, Haoming Wu, Xin Yong, Yao Wang, Shuhao Yang, Diyi Fan, Yibo Xiao, Lanyu Che, Kun Shi, Kainan Li, Chengdong Xiong, Huili Zhu, Zhiyong Qian

**Affiliations:** ^1^ Clinical Medical College and Affiliated Hospital of Chengdu University Chengdu University Chengdu Sichuan China; ^2^ Department of Biotherapy Cancer Center and State Key Laboratory of Biotherapy West China Hospital Sichuan University Chengdu Sichuan China; ^3^ Key Laboratory of Birth Defects and Related Diseases of Women and Children, Department of Paediatrics, West China Second University Hospital, State Key Laboratory of Biotherapy and Collaborative Innovation Center of Biotherapy Sichuan University Chengdu Sichuan China; ^4^ Department of Orthopedics The First Affiliated Hospital of Chongqing Medical University Chongqing China; ^5^ University of Chinese Academy of Sciences Bei Jing China; ^6^ Department of Reproductive Medicine, Key Laboratory of Birth Defects and Related Diseases of Women and Children of Ministry of Education West China Second University Hospital of Sichuan University Chengdu Sichuan China

**Keywords:** biomaterial, endometrial repair, endometrium regeneration, menstrual cycle, regenerative medicine, tissue engineering scaffolds

## Abstract

The endometrium is a unique human tissue with an extraordinary ability to undergo a hormone‐regulated cycle encompassing shedding, bleeding, scarless repair, and regeneration throughout the female reproductive cycle. The cyclical repair and regeneration of the endometrium manifest as changes in endometrial epithelialization, glandular regeneration, and vascularization. The mechanisms encompass inflammation, coagulation, and fibrinolytic system balance. However, specific conditions such as endometriosis or TCRA treatment can disrupt the process of cyclical endometrial repair and regeneration. There is uncertainty about traditional clinical treatments' efficacy and side effects, and finding new therapeutic interventions is essential. Researchers have made substantial progress in the perspective of regenerative medicine toward maintaining cyclical endometrial repair and regeneration in recent years. Such progress encompasses the integration of biomaterials, tissue‐engineered scaffolds, stem cell therapies, and 3D printing. This review analyzes the mechanisms, diseases, and interventions associated with cyclical endometrial repair and regeneration. The review discusses the advantages and disadvantages of the regenerative interventions currently employed in clinical practice. Additionally, it highlights the significant advantages of regenerative medicine in this domain. Finally, we review stem cells and biologics among the available interventions in regenerative medicine, providing insights into future therapeutic strategies.

## Graphical abstract

This image summarizes diseases and therapeutic interventions for cyclic endometrial repair and regeneration. We summarize endometrial repair at different menstrual phases and the external factors contributing to endometrial repair imbalances. Current research advances in endometrial repair are outlined. Examples include hormone therapy, stem cell therapy, and so on.

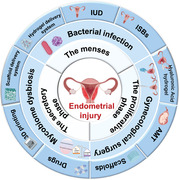

## 1 INTRODUCTION

The endometrium, the mucosal layer of the uterus, is hormonally regulated, experiencing cycles involving shedding, bleeding, scarless repair, and regeneration.^1^, ^2^ Typically, each cycle in a female reproductive life span approximately 1 month. It exhibits high dynamism and sustains continuous cell turnover throughout the reproductive period. A cycle can be subdivided into a menstrual phase, a proliferative phase, and a secretory phase. During the menstrual phase, the endometrium undergoes shedding in fragments, accompanied by bleeding.^3^, ^4^ The proliferative and secretory phases are dedicated to endometrial repair and oocyte secretion. These repair and regeneration processes are associated with cyclical changes in the female endometrium. The process of regeneration includes endometrial epithelialization, glandular regeneration, and vascularization.^5^, ^6^, ^7^ Mechanisms encompassed in the repair and regeneration of the endometrium involve a balance of inflammation, coagulation, and fibrinolytic systems.^8^, ^9^, ^10^, ^11^

Congenital anomalies and acquired endometrial repair disorders, including diminished hormone levels, uterine fibroids, and scar formation postprevious caesarean section or myomectomy, may disrupt the cyclical endometrial repair and regeneration process.^12^, ^13^ This disruption subsequently influences the uterine cavity, contractility, and the process of embryo implantation, ultimately leading to uterine factor infertility, miscarriage, functional uterine bleeding, pelvic pain, leukorrhea, and other obstetrical complications.^14^ The rising prevalence of clinical cases highlights the impact of diverse disorders on endometrial regeneration throughout the menstrual cycle.^15^, ^16^ These disorders encompass menstrual irregularities, endometriosis (EMS), fibroids, and endometrial polyps.^17^, ^18^ Presently, therapeutic approaches to manage these conditions encompass medication for maintenance and surgical excision of aberrant tissue.^19^ The central premise of its treatment strategy is to promote endometrial repair and regeneration. Methods available to promote endometrial repair after surgery are IUDs, intrauterine suitable balloons (ISBs), Foley catheters, hyaluronic acid hydrogels (HA gels), cross‐linked HA gels, estrogens, drugs, growth factors, and amniotic membranes.^20^, ^21^, ^22^ Some methods have shown good therapeutic results in clinical trials. However, they are difficult to adapt to different patient situations. We will show the disadvantages of these methods later. Nowadays, maintaining postoperative endometrial repair and regeneration poses new challenges.

Regenerative medicine is a cutting‐edge and innovative field of research aimed at repairing or replacing the body's dysfunctional cells, tissues, or organs.^23^, ^24^ By researching the organs' normal tissue characteristics and functions, wound repair, and regeneration mechanisms, we are searching for effective biological therapies to promote the self‐repair and regeneration of the organism.^25^, ^26^ Its goal is to maintain, repair, regenerate, or improve the function of injured tissues and organs. The field encompasses four areas: cell therapy, tissue engineering, biomaterials science, and autologous and allogeneic transplantation.^27^, ^28^, ^29^, ^30^, ^31^ Research in this field advances rapidly and incorporates techniques like tissue‐engineered scaffolds (e.g., bionic scaffolds using biodegradable, resorbable materials to facilitate tissue repair) and creating complex three‐dimensional tissues through bioprinting.^32^, ^33^, ^34^, ^35^ These rapid advancements provide research directions and potential treatments for numerous complex diseases. Illustrative cases comprise intrauterine adhesions (IUAs), EMS, and abnormal uterine bleeding.

Due to materials science, clinical medicine, and life sciences advancements, regenerative medicine has exhibited exceptional promise in endometrial regeneration. A particularly crucial aspect involves employing biocompatible scaffolds to deliver stem cells or bioactive substances to injured tissues, expediting endometrial regeneration.^36^

This review comprehensively examines the established processes of cyclical endometrial repair and regeneration, delves into the mechanisms underpinning dysfunction, and explores a range of disorders and therapeutic interventions pertinent to endometrial regeneration. Furthermore, it emphasizes the utilization of regenerative medicine in the context of endometrial regeneration, encompassing stem cell therapy and tissue engineering. Additionally, the review presents prospective approaches to uphold endometrial regeneration homeostasis, encompassing physical barriers, growth factor/cell carrier strategies, and 3D printing techniques. Furthermore, it highlights their advanced applications in the context of endometrial repair and regeneration.

## 2 PHASING OF THE MENSTRUAL CYCLE AND ENDOMETRIAL REPAIR: EXPLORING THE MECHANISMS OF REPAIR AT DIFFERENT OUTCOMES

Cyclical endometrial repair and regeneration is inextricably linked to the female reproductive system, in which the endometrium dominate. We categorize cyclical endometrial repair and regeneration into three scenarios. These are regular menstrual cycle repair, deficient repair, and excessive repair.

### 2.1 Overview of endometrium

The female reproductive system consists mainly of the vulva and vagina at the bottom lower end and the cervix, uterus, fallopian tubes and ovary at the upper end. The most important parts are the endometrium, which supports the development of the zygote. Additionally, the endometrium is influenced by the ovaries, which produce ovarian follicles and estrogen.^37^, ^38^ Both maintains the cyclical repair and regeneration of the endometrium, thus conferring the female reproductive function.

The endometrium can be divided into two layers: the basal layer and the functional layer. The surface 2/3 of the endometrium is the functional layer, mainly affected by ovarian sex hormones, which undergo cyclic changes and shedding. The basal layer consists mainly of glands containing the stroma, the basal part, the supporting vascular system and various immune cell populations such as natural killer cells (NK cells), neutrophils, macrophages, and lymphocytes (T or B cells).^39^, ^40^ Remains complete during menstruation and provides a source of cellular production. It also renews the functional layer after menstruation.^41^

The endometrium has many glands that secrete mucus and lubricating substances on its surface. These secretions help reduce friction within the uterine cavity and support the implantation of the zygote and the development of the embryo. If the zygote is successfully deposited, the endometrium provides nutrients and supports embryo's growth. During the second half of embryonic development, trophoblast cells invade the myometrium and form a specialized organ, the placenta. During pregnancy, the placenta is the site of nutrient exchange between the fetus and the mother. It is also a vital endocrine organ that participates in fetal development and maternal adaptive responses by producing a variety of hormones.^42^, ^43^

Reproduction is integral to human progress. Statistics reveal that within the past 40 years, the average number of children per woman has decreased by 40%.^44^, ^45^ According to statistics, 8−12% of couples worldwide are affected by infertility, the majority of which is caused by defects in the functioning of the endometrium.^46^, ^47^ The prevalence of female infertility increased by 0.370% per year during 1990−2017, much higher than 0.291% for men.^48^ The most common cause of female infertility is embryo implantation failure.^49^, ^50^, ^51^ The multiple causes of infertility have led to a focus on maintaining cyclic endometrial repair. In the next section, we will summarize the phases menstrual cycle.^52^

### 2.2 The menstrual cycle and stable endometrial repair and regeneration

In women of childbearing age, the growth of the ovarian follicles, ovulation, and the corpus luteum formation is accompanied by the cyclic secretion of estrogen and progesterone, which ultimately causes the cyclic shedding of the endometrium and bleeding.^12^, ^53^, ^54^ This phenomenon is called menstruation. Menstruation usually occurs once a month, and the menstrual cycle begins on the day of the first visible bleeding (day 1). The cycle length varies from person to person, usually 21−35 days, with an average of 28 days.^55^ Based on the changes in the menstrual cycle and the shape and function of the endometrium, we categorize the menstrual cycle into three phases: menstrual, proliferative, and secretory.

#### 2.2.1 The menses

The menses is usually the first few days of the start of menstruation (∼days 1−5), and overlap with part of the proliferative phase can occur. In this part of the period, because there is no zygote implantation occurring, the corpus luteum undergoes atrophy, leading to a decrease in the levels of estrogen and progesterone and spasmodic constriction of some of the small arteries of the endometrium.^56^ Thus, ischemia, deformation, and necrosis occur in the two‐thirds of the endometrium close to the luminal surface, ultimately shedding and rupturing blood vessels, causing hemorrhage.^57^, ^58^

During the menses phase, the endometrium is shed in fragments, and at the same time, rupture and rapid repair occur in adjacent areas.^59^, ^60^ Re‐epithelialized repair of the endometrium occurs during the menstrual phase and is completed by the cessation of bleeding (3–5 days). Endometrial repair differs from skin repair in that ordinary course endometrial repair does not cause scarring. It is manifested by rapid re‐epithelialization of the endometrial surface.^61^

In the early stages of endometrial repair, wound clearance and repair coincide, and it is difficult to sort out individual immune cell types.^61^, ^62^ However, we believe that leukocytes and other immune cells enter the injury site early to remove dead cells and tissue residues. This clearance process is essential for new endometrial regeneration. A decrease in neutrophil and CXCL4^+^ macrophages has been shown to delay endothelial repair in mouse models.^62^, ^63^ Although there are significant differences between humans and mice, both cell types likely positively affect endometrial repair. Re‐epithelialization begins with migrating epithelial cells from adjacent intact epithelial tissue or endothelial glands to the injury site.^42^ This stage is accompanied by dynamic changes in the extracellular matrix (ECM), but its purpose is to accelerate the rate of cell migration. It is reflected in changes in basement‐membrane proteins, integrins and cell‐adhesion molecules. Accompanying the re‐epithelialization of the endometrium is the regeneration of subepithelial stroma and spiral arterioles. Both are more severely injured during endometrial shedding.^59^, ^60^ Regenerating endometrial epithelial cells, macrophages, and mesenchymal stromal cells produce vascular endothelial growth factor (VEGF) and basic fibroblast growth factor (bFGF) to promote angiogenesis.^64^, ^65^

Re‐epithelialization of the endometrium, glandular regeneration, and revascularization is essential for endometrial repair. For the lack of scarring during endometrial repair, Salamonsen et al.^61^ attributed it to the multiple compositions of menstrual fluid and retention of the basal layer. When the basal layer is injured, the endometrial repair is disrupted, and excessive endometrial regeneration may be induced, further compromising the normal uterine function (discussed subsequently).

#### 2.2.2 The proliferative phase

The proliferative phase, also known as the follicular phase, is usually defined as the time from the beginning of the menstrual period to ovulation and corresponds to the period of rapid follicular growth during cyclic recruitment.^66^ The rapid follicular development during this phase is accompanied by an increase in estrogen secretion so that the endometrium injured during menstruation is gradually further repaired, growing and thickening; at the same time, the number of glands increases, and the spiral arterioles lengthen and enlarge, as well as bend.^67^ Repair during the proliferative phase is inextricably linked to estrogen, stem cells, and glands.

As early as 1989, it was proposed that stem cells are located in the endometrial basal layer and participate in the cyclic regeneration of the endometrium.^68^ Subsequent endometrial‐related studies have identified various cells with classical stem cell properties, such as self‐renewal and differentiation in vitro, including epithelial progenitors and endometrial mesenchymal stem cells (eMSCs).^69^, ^70^ Fortunately, some studies have demonstrated that these stem cells can accelerate endometrial repair, such as the scaffold‐carrying eMSCs mentioned later. In the 1980s, it was found that estrogen affects the cells at the base of the glands, stimulating them to proliferate and migrate into the cavity.^71^ Following this, it was discovered that vascular regeneration is also inextricably linked to estrogen. However, the molecular mechanisms of both regeneration and repair remain to be investigated.

#### 2.2.3 The secretory phase

The secretory phase is also known as the luteal phase. It is usually defined as the period from the time after ovulation until the onset of the next menstrual period. During this phase, the corpus luteum, formed after ovulation, secretes large amounts of progesterone and estrogen, and the thickness of the endometrium further increases. In contrast, the secretory function of the endometrium is enhanced. It is manifested by the fact that the glands become more curved and secrete a large amount of mucus. Small vesicles containing glycogen appear at the glandular epithelial cells' base. It should be noted that the spiral arterioles also grow more curved.^72^, ^73^ Ciliated and nonciliated epithelial cells with transcriptional activation can be observed in glandular and luminal epithelium sites.^6^ These changes in the glands mainly focus on altering the adhesion capacity and facilitating embryo implantation. The endothelial stroma becomes edematous at this stage. At the same time, the spindle‐shaped stromal cells enlarge and become rounded, differentiating into predecidual cells. These cells, as well as the changes in the spiral arterioles, provide the basis for fertilization.

Before ovulation, the production of estrogen causes the cervical mucus to be clear and thin, which facilitates the passage of sperm. After ovulation, progesterone causes the cervical mucus to become thick and sticky, which prevents the passage of sperm. The process of interaction and invasion of the follicle with the endometrium is called implantation.^74^, ^75^ For implantation to occur, the endometrium must undergo morphologic and functional changes to become receptive to the embryo. The morphological hallmark of this stage is decidua formation.^76^, ^77^ When the endometrium is injured and its repair is impaired, it cannot undergo decidua formation, leading to difficulties in implantation and infertility.

### 2.3 The role of estrogen and progesterone

Estrogen consists of estrone, estradiol, and estriol. Of the three, estradiol is the most active. It is secreted mainly by granulosa cells and endometrial cells before ovulation and by the corpus luteum after ovulation.^78^ If fertilization occurs, it is produced mainly by the corpus luteum and placenta after pregnancy. Its effects include promoting endometrial thickening and glandular proliferation.^79^, ^80^ It also promotes vascular regeneration, mainly related to VEGF production. The rest of the physiological effects include increasing cervical mucus production and thinner, facilitating fertilization.^81^, ^82^ It also promotes the proliferation and keratinization of the vaginal epithelium.

Progesterone includes progesterone, 20 α‐hydroxyprogesterone, and 17 α‐hydroxyprogesterone. Of these, progesterone is the most active. It mainly thickens the endometrium further and accelerates the decidua formation in endometrial stromal cells.^83^ It is mainly used to stabilize the endometrium during pregnancy and maintain pregnancy.^84^

Estrogen is more widely used in the field of endometrial repair than progesterone. Estrogen can accelerate the endometrial repair process more directly. It has also undergone a long period of research and clinical trials and has relatively few side effects and risks. In contrast, progesterone may cause breast tenderness, osteoporosis, and so on. These provide a theoretical basis for our subsequent studies on endometrial repair.

### 2.4 Unbalanced repair and regeneration of the endometrium

**FIGURE 1:**
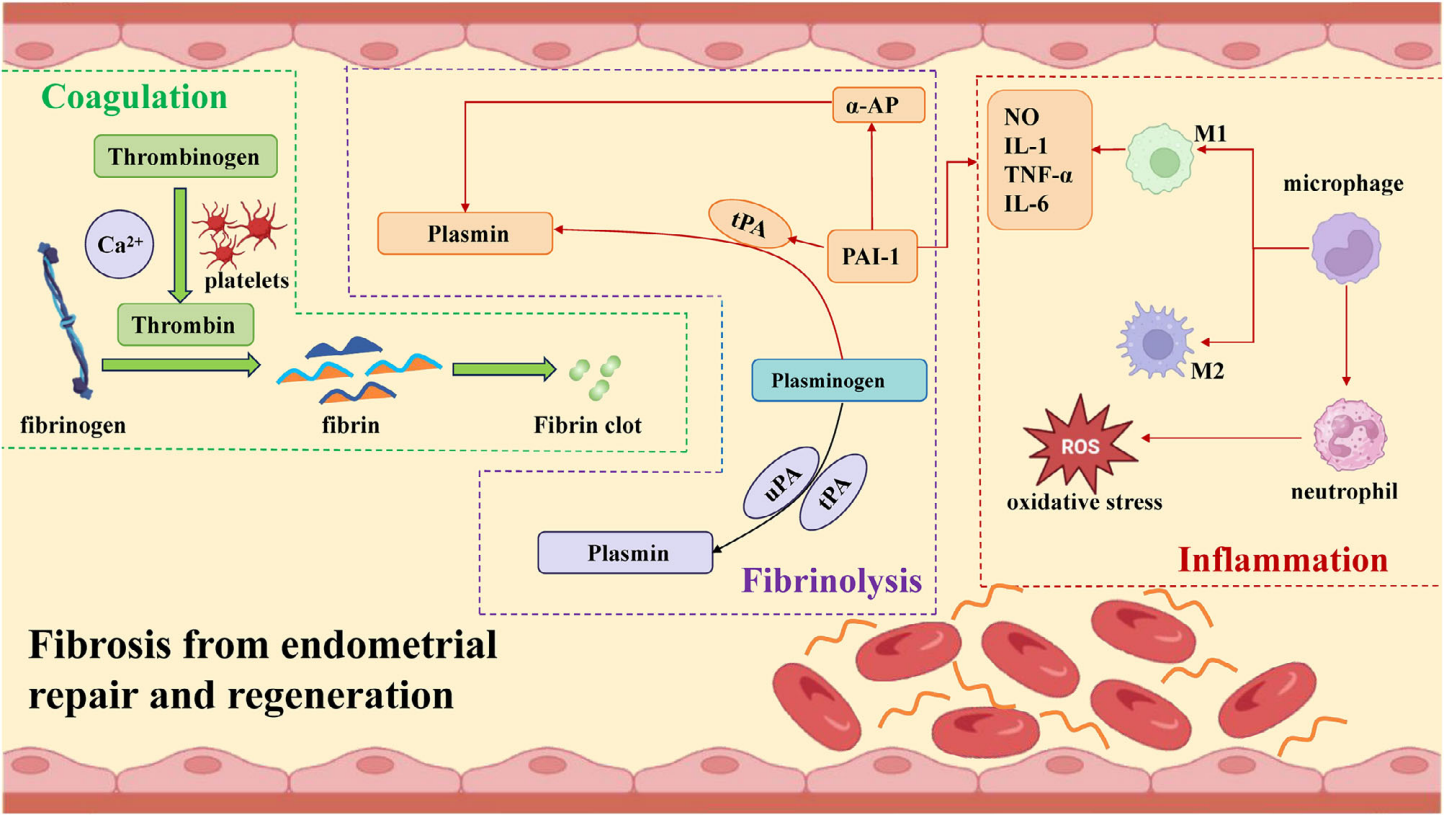
Schematic diagram on the interaction among coagulation, inflammation, and fibrinolytic systems in endometrial repair and regeneration. In endometrial repair and regeneration, upregulation of cytokine levels activates the NF‐κB signaling pathway, which ultimately leads to an increase in the expression of plasminogen activator inhibitor (PAI‐1), thereby inhibiting the activity of the fibrinolytic system. At the same time, if the balance between tPA and PAI‐1 is disrupted after sudden acute injury to the endometrium, it will lead to fibrin leakage and persistent fibrin aggregation.

Unbalanced repair of the endometrium after shedding can be divided into two types: poor endometrial repair and excessive endometrial repair. Our response to the former tends to be postoperative estrogen and regular monitoring. The latter often results in fibrosis. The mechanism of fibrosis resulting from different degrees of endometrial shedding is not precise. Combining the numerous research and comparing them with abdominal diseases, we believe that the mechanism by which endometrial hyperplasia induces adhesions may involve coagulation, inflammation, and fibrinolysis (Figure 1).

#### 2.4.1 Coagulation and inflammation

When the endometrium is shedding, the first manifestation is vasoconstriction near the injury, mainly to reduce the blood supply and relieve bleeding. At the same time, the platelets are activated, mainly in the form of adhesion, aggregation, and release.^85^ Platelets release 5‐HT and TXA2, which further constrict the blood vessels. Then comes the most crucial activation of the coagulation system. The essential hemostatic function in the coagulation system is the prothrombin complex, which consists mainly of coagulation factor X and coagulation factor V. Its action is to convert the thrombinogen into thrombin, which can subsequently convert fibrinogen into fibrin.^86^, ^87^ Fibrin can clot with platelets to form a clot and perform hemostatic functions.

The coagulation pathway is often associated with inflammation. When sentinel cells such as macrophages recognize necrotic tissue, they secrete inflammatory mediators (represented by histamine) to increase vascular permeability and facilitate infiltration of the wound by inflammatory cells (represented by neutrophils and macrophages). When an acute inflammatory response occurs, fibrinogen increases sharply. During this phase, IL‐6 secreted by M1 macrophages plays a crucial role in fibrin. It promotes fibrinogen synthesis, while a decrease in fibrin upregulates IL‐6 levels.

Before menses, a decrease in progesterone causes an upregulation of the levels of certain specific chemokines, commonly CXCL‐8, CX3CL1, and CCL22.^88^, ^89^ They bind to receptors on leukocytes and promote the expression of adhesion molecules.^90^ Accompanied by decreased neutrophil levels, monocytes enter the designated site and promote wound debridement. Many inflammatory cells are present during menstruation, which contribute to the shedding of the endothelium and potentially initiate the repair process.^91^ For example, in a mouse model, eliminating neutrophils affects endometrial shedding and significantly delays endometrial repair.^92^ The persistence of leukocytes leads to hyper‐fibrosis.^93^

After the acute endometrial shedding, exudated inflammatory cells can upregulate cytokine levels, mainly in elevated levels of TNF‐α and IL‐1. Following this, M1 macrophages accumulate in the wound, releasing TNF‐α, IL‐1, and IL‐6. Cytokines involved in wound healing activate the NF‐κ B signaling pathway and promote the secretion of a downstream inflammatory factor. This may eventually lead to increased expression of plasminogen activator inhibitor (PAI‐1), which inhibits the activity of the fibrinolytic system.^94^, ^95^ In this process, activated neutrophils and macrophages may be associated with oxidative stress that promotes adhesion formation, and Torres et al.^94^ suggest a corresponding view of the related mechanism. Oxidative stress leads to excess toxic substances that lyse endothelial cells and fibroblasts. When cellular contents flow out, adhesion formation is exacerbated.^96^

#### 2.4.2 Coagulation and fibrinolysis

When the endometrium is in a state of rapid shedding, the ECM will undergo massive proliferation to repair the defective endometrium. Fibrin is usually transient during endometrial regeneration and is degraded by the fibrinolytic system after healing. If the fibrinolytic system is impaired and fibrin is challenging to break down, fibroblasts will produce collagen with ECM proteins developing adhesions.^97^

The fibrinolytic system includes plasminogen, plasmin, plasminogen activators (tPA), and urokinase type plasminogen activator (uPA). We need to note that inhibitors are necessary for the dissolution equilibrium. Inhibitor systems include PAI‐1 and α‐antiplasmin (α‐AP). Under normal conditions, tPA and uPA can activate the precursor plasminogen into plasmin and promote fibrinolysis. tPA is regulated by the fibrinolytic system's PAI‐1.^98^ tPA‐1 can promote the proliferation of adipocytes and stimulate the inflammatory response of macrophages, inducing the development of adhesions, which is inhibited by the production of α‐AP by the liver.^99^

Accompanying acute endometrial injury, the balance between tPA and PAI‐1 is disrupted, leading to fibrin exudation and persistent fibrin aggregation. At the same time, the previously mentioned increased inflammatory response (upregulation of IL‐1, IL‐6, and TNF‐α levels) can downregulate tPA activity, reduce fibrinolytic system function, and ultimately promote adhesion formation. Similarly, elevated fibrinogen and fibronectin upregulate TNF‐α and IL‐1β levels, which stimulate the release of PAI‐1 from mesothelial and endothelial cells, inhibit fibrinogen activation, and promote adhesion development.^100^, ^101^

Regardless of the cause of endometrial shedding, maintaining a balance between endometrial repair and regeneration is critical and necessary. Adjunctive endometrial repair is often performed clinically after some gynecological procedures. However, in most cases, doctors choose to ignore it. In the case of abortion, for example, we understand that in southwest China, some hospitals will selectively perform postoperative endometrial repair treatment, the interventions being mainly up to the patient. However, in some hospitals in the north, the consequences of the procedure, including IUAs, are not mentioned to patients at all. Some hospitals provide information about IUAs, while others ignore them. Most hospital patients are diagnosed with infertility due to IUA before undergoing transcervical resection of adhesion (TCRA), followed by adjuvant therapy to restore the normal function of the endometrium. Therefore, most subjects in clinical research are patients diagnosed with IUA, while nonclinical studies prefer models with endometrial injury. For endometrial repair and regeneration, our observational indices are mainly endometrial thickness, number of glands and vascular regeneration. However, inhibition of fibrosis, prevention of adhesion recurrence, reduction of fibrotic area and improvement in embryo pregnancy rate are important manifestations of regular endometrial regeneration. We hope that more and more researchers and doctors will focus on endometrial repair and regeneration to prevent female infertility at the source and to improve pregnancy outcomes in patients.

## 3 DISEASES AND CLINICAL REGENERATIVE INTERVENTIONS IN CYCLICAL ENDOMETRIAL REPAIR AND REGENERATION

Previously, we introduced the normal female menstrual cycle and the importance of estrogen and progesterone in endometrial repair. However, in clinical practice, we find that more patients have a variety of gynecologic diseases that can lead to endometrial injury or impaired repair. This section will summarize the repair and regeneration disorders and the corresponding clinical interventions.

### 3.1 Diseases in cyclical endometrial repair and regeneration

Numerous diseases cause abnormal endometrial shedding, which we have broadly categorized into microbial‐type diseases and diseases associated with abnormal tissue in the uterine cavity. The former includes infections by foreign microorganisms and diseases caused by opportunistic pathogens. Representatives of the latter include EMS, fibroids, and IUAs. These disorders' effect on endometrial regeneration often interferes with cyclical endometrial repair and regeneration and even with the patient's reproductive system function. Common symptoms include abnormal endometrial shedding, bleeding, amenorrhea, and infertility. They are treated with hysteroscopic tissue removal, a surgery that often causes injury to the basal layer, which in turn leads to the patient's inability to self‐repair to maintain the normal morphology of the endometrium, usually resulting in a lack of regeneration or over‐repair of the endometrium. Maintenance of endometrial repair and regeneration after surgery is deficient in current clinical practice. We will summarize this in the clinical intervention section.

**FIGURE 2:**
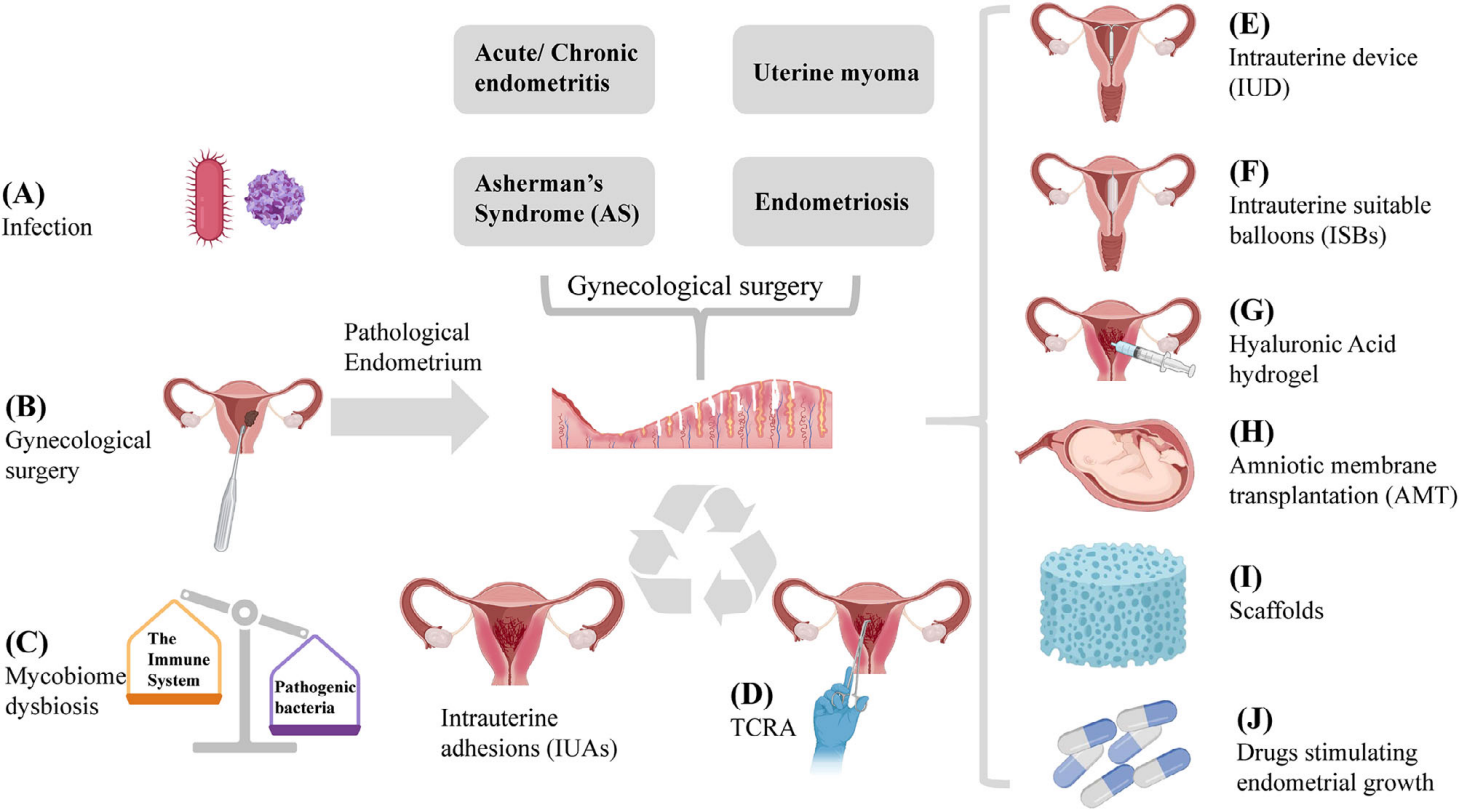
Diseases and clinical interventions of cyclical endometrial repair and regeneration. (A) endometrial infection, (B) gynecological surgery, and (C) mycobiome dysbiosis, are the main causes of infection of microorganisms. Abnormal regenerative diseases include abnormal fibroplasia, abnormal smooth muscle proliferation, and abnormal endometrial growth. The resolution of their postoperatively induced intrauterine adhesions is (D) TCRA. Measures to promote endometrial repair and regeneration is followed by (E) intrauterine device (IUD), (F) intrauterine suitable balloons (ISBs), (G) hyaluronic acid hydrogel, (H) amniotic membrane transplantation (AMT), (I) uterine scaffolds to reserve the space of the uterus, and (J) drugs stimulating endometrial growth.

Few people view abnormal endometrial shedding, intrauterine surgery, and disease as a whole. For example, during the abortion, scraping is performed, which is very likely to cause injury to the endometrium's basal layer, inducing IUAs. After the adhesions occur, we will perform TCRA, and even though we will perform postoperative endometrial repair treatment, the recurrence rate is as high as 20−62.5%.^102^ Common gynecologic conditions additionally include uterine myoma, EMS, and uterine polyps. Their standard treatment methods include medication combined with hysteroscopic surgery (Table 1). Surgeries in different uterine sites can cause varying degrees of shedding to the basal layer of the endometrium. If the endometrial repair process is abnormal, leading to endometrial adhesions or fibrosis, there is a high probability of partial or total occlusion of the uterine cavity, eventually causing infertility. It ultimately comes back to the occurrence of IUAs (Figure 2).

**TABLE 1:** Causative factors, pathological manifestations, diagnostic measures, clinical presentations, and treatments of endometrium disorders.^a^

Endometrial disorder	Causative factors	Pathological manifestations	Diagnostic measures	Clinical presentations	Treatments	References
Infection of microorganisms	Bacteria, biological virus, fungi, inflammation of the basal layer, placental residues, intrauterine devices, uterine fibroids	The endometrium is congested, edematous or necrotic, purulent discharge from the endometrial surface, diffuse infiltration of neutrophil granulocytes, destruction of the endometrial mesenchyme	Histopathological examination, hysteroscopy, white belt examination, microbiological examination, complete blood count	Fever, lower stomach pain, increased leucorrhea, tenderness in the uterus; pelvic pain, difficulty with intercourse, intrauterine adhesions	Cervical conetomy or total hysterectomy (generally not used), antibiotic therapy (doxycycline, metronidazole, ciprofloxacin)	^103^, ^104^, ^105^, ^106^, ^107^, ^108^, ^109^, ^110^, ^111^, ^112^, ^113^, ^114^, ^115^
Intrauterine adhesions	Repeated abortions, gross infections, microecological imbalance in the uterine cavity	Injury to the basal (partial/complete) layer of the endometrium and formation of fibrous connective tissue hyperplasia, formation of endometrial scarring, fibrosis, anatomical formation of adhesions	Hysteroscopy (gold standard), ultrasound: 2D or 3D‐TV US, SHG	Periodic abdominal pain, abnormal menstruation, low fertility, recurrent abortions; retained placenta, postpartum hemorrhage, preterm birth, intrauterine adhesions	TCRA, estrogen, (anti‐inflammatory) drug therapy, stem cell therapy, hyaluronic acid (HA), amniotic membrane	^116^, ^117^, ^118^, ^119^, ^120^, ^121^, ^122^, ^123^, ^124^, ^125^, ^126^
Endometriosis	Reflux of menstrual blood into the uterine cavity, endocrine hormone imbalance, genetic	Ectopic endometrium is often diffused throughout the uterus, proliferation of fibrous tissue and muscle fibers, Ectopic cells are scattered in the muscle layer or concentrated in a certain area	Antiendometrial antibody (EMAb), hysteroscopy, B‐mode ultrasonography	Abdominal pain, abnormal menstruation, infertility, intrauterine adhesions	Curettage, Mirena, analgesic, antibiotic therapy, hormone therapy	^127^, ^128^, ^129^
Thin endometrium	Severe endocrine disorders, abortion, abnormal uterine development, repeated curettage	Reduced VEGF expression; vascular dysplasia; thin endometrial thickness	Hysteroscopy, ultrasonography, endometrial biopsy	Shorter menstrual period, decreased rate of fertility, increased rate of miscarriage, intrauterine adhesions	Stem cell therapy, hormone therapy	^130^, ^131^, ^132^, ^133^, ^134^, ^135^, ^136^, ^137^
Endometrial polyps	Severe endocrine disorders, long‐term gynecological inflammation, intrauterine foreign device (e.g., contraceptive device), abortion, puerperal infection	Endometrial polyps can be single or multiple, round, oblong, or tongue‐shaped in appearance, with a minimum diameter of only 1–2 mm and occasionally as large as several centimeters.	Ultrasound scanning by vagina, hysteroscopy, histopathological examination	Metrorrhagia, abdominal pain, infertility	Transcervical resection of endometrium, dilatation and curettage of uterine, hysterectomy, hormone therapy	^138^, ^139^, ^140^, ^141^, ^142^

^a^ TV US, transvaginal ultrasound; SHG, sonohysterography; TCRA, transcervical resection of adhesions; BMDSC, bone marrow‐derived stem cell; VEGF, vascular endothelial growth factor; Mirena, Mirena is a hormonal intrauterine device (IUD) that provides long‐term prevention.

The assessment of endometrial repair and regeneration is diverse. In the case of post‐TCRA, for example, we need to regularly record the patient's endometrial thickness, menstrual status (volume and color of menstruation), postoperative bleeding, adhesions, and postoperative complications. For patients with reproductive intentions, we will count the corresponding pregnancy rates. In nonclinical studies, we record more data, including the number of glands in the regenerative endometrium, the density of blood vessels, the number of fibroblasts, and the levels of different inflammatory factors.

#### 3.1.1 Infection of microorganisms in cyclical endometrial repair and regeneration

Microbial diseases refer to endometrial infection by bacteria, viruses, or other microorganisms. It can cause various diseases and conditions, including endometritis, pelvic inflammatory disease, and salpingitis, of which endometritis is the most common.^143^, ^144^ Endometritis is an inflammatory disease of the endometrium. From the histopathology and the duration of the disease, it can be divided into two types: acute endometritis and chronic endometritis (CE).^145^ Both have different clinical symptoms and effects on endometrial regeneration.

##### Acute endometritis

Acute endometritis is a common gynecopathy disease usually caused by bacterial infections such as staphylococcus, *Escherichia coli*, streptococcus, and anaerobic bacteria.^146^ Inflammation causes swelling, redness, ulceration, and bleeding of the endometrial tissue, causing patients to suffer from back pain, abdominal pain, increased vaginal discharge, and fever. The pathological features are microabscesses formation and neutrophil tissue infiltration.^147^, ^148^ The clinical diagnosis includes a complete blood count, histological section, and hysteroscopy. For its treatment, antibiotic therapy is the mainstay and auxiliary curettage. Direct bacterial invasion or toxin production injures the endometrium, resulting in inflammation of the endometrial tissue. The endometrial cells and tissues are continuously injured.

Antibiotic therapy is the mainstay for therapeutic measures, with curettage as an adjunct. Bacteria invade directly or produce toxins that injure the endometrium, causing the endometrial tissue to become inflamed. Endometrial cells and tissues are constantly being injured. The effects on cyclic endometrial regeneration and repair include several aspects: first, inflammation‐induced destruction of the basal layer can leave the endometrium weak, irregular, or continually injured, interfering the normal regeneration process. Second, the inflammation response may lead to scar tissue and adhesion zones forming, which affects the regular menstrual blood elimination and estrogen secretion, leading to a blockage of the regeneration process. Third, continued endometrial inflammation can cause inflammatory cells and inflammatory cytokines to be present in the uterine cavity for an extended period, thus interfering with the normal regeneration of the endometrium.

Currently, the clinical treatment of acute endometritis is not associated with promoting endometrial and regeneration, and we hope that more attention will be paid to this aspect in the future.

##### Chronic endometritis

Unlike the former, the subtle symptoms of CE are usually overlooked by physicians and patients. It is a chronic inflammatory disease of the pelvis with accompanying pathological manifestations such as superficial endometrial edematous, high density of mesenchymal cells, and endometrial interstitial plasma cell infiltration.^149^ The relevant diagnostic tests are histopathological examination and hysteroscopy. Before this, increasing data suggested an increasingly strong association between CE and infertility. For example, 28% of patients with infertility of unknown etiology have CE, while 14−41% of patients with repeating implantation failure (RIF) have CE.^150^, ^151^, ^152^ Clinical evidence for a causal relationship between CE and reproductive dysfunction remains to be established. Nevertheless, we hypothesize that CE is inextricably linked to impaired endometrial regeneration and hope that the interventions described below will treat CE and maintain regular endometrial repair and regeneration.

#### 3.1.2 Diseases associated with abnormal tissue in cyclical endometrial repair and regeneration

Diseases that interfere with cyclic endometrial regeneration and repair are often accompanied by abnormal regenerative tissue. These include abnormal fibroplasia, abnormal smooth muscle proliferation, and abnormal endometrial growth. The clinical solution is centered on hysterectomy tissue, with postoperative estrogen administration as an adjunct. Table 1 describes the pathophysiology, diagnosis, and treatment strategies for various disorders that interfere with endometrial repair and regeneration.

##### Intrauterine adhesions

IUAs, also known as Asherman's syndrome, is characterized by scarring and adhesions in the pelvic or intrauterine cavity.^153^ The disease process is usually characterized by multiple pathways of injured to the endometrial basal layer, leading to imbalances in endometrial regeneration and ultimately to disturbances in the fibrinolytic system and fibrosis^154^ (Figure 2). Clinical manifestations are cyclic abdominal pain, abnormal menstruation and low fertility, including recurrent miscarriage, retained placenta, postpartum hemorrhage, and preterm labor.^124^ According to reliable reports, there are 36–53 million pregnancy terminations annually worldwide, of which approximately 90% occur in the early stages of pregnancy.^155^ The incidence of IUA following early abortion is approximately 6.3%14. Therefore, IUA is an essential public health problem for female fertility.

IUA can be confirmed and graded by hysteroscopy.^156^ The standard treatment for mild IUAs is TCRA, which involves the removal of adhesive tissue and the separation of the uterine cavity. Treatments of moderate to severe IUAs are often: TCRA + postoperative antiadhesion material + medication (Figure 2). Recovery after TCRA is mainly determined by the density of blood vessels and the number of glands.^157^ Injury to the normal endometrium can occur during the TCRA and needs to be noted and avoided in the clinical surgery. Despite the variety of postoperative adjuvant treatments available in the clinic, data suggest that patients with severe IUAs have a recurrence rate of IUA as high as 20−62.5% after TCRA. Therefore, preventing the recurrence of postoperative adhesions is an urgent problem that needs to be solved.

When the basal layer is injured, the regenerative capacity of the endometrium and the vitality of the corresponding small blood vessels are reduced, and various immune cell populations cannot perform their normal functions. At the same time, fibrous tissue proliferates, eventually leading to endometrial fibrosis. When the degree of fibrosis is high enough, the uterine cavity/cervical canal is wholly or partially lost and the uterus cannot perform its regular physiological functions, resulting in difficulty in conception.^158^

##### Uterine fibroid

Uterine fibroid, or uterine leiomyomas (UL), are substantial tumors composed of uterine smooth muscle, connective tissue, and glands and are the most common benign gynecologic tumors in women of childbearing age. UL can cause pelvic pain, infertility, miscarriages, and other adverse pregnancy outcomes.^159^, ^160^ According to the recommendations of the International Federation of Gynecology and Obstetrics (Figure 2), uterine fibroids can be classified into nine types.^161^

Fibroid of varying degrees can put pressure on the endometrium, distort the shape of the uterine cavity and increase irregular shedding of the lining. Management of fibroids includes observation, medication, surgical, and nonsurgical treatments.^162^, ^163^ Surgical treatment includes myomectomy, hysterectomy, and endometrial resection. For postoperative treatment of uterine fibroids, we found that the core is to promote endometrial repair and regeneration. Treatments include estrogen and HA gel (if accompanied by laparotomy, laparotomy antiadhesion measures are performed). In a recent study after hysteroscopic myomectomy, HA gel reduced the production of fibrotic tissue.^164^ For now, there is still room for improvement in postoperative endometrial repair. For example, different types of uterine fibroids correspond to different endometrial repair strategies. However, the ideal conditions for clinical treatment have yet to be achieved.

##### Endometriosis

EMS is a common condition in women of childbearing age, with a 10–15% prevalence. The prevalence in women with combined pelvic pain or infertility can be 20–60%.^165^ EMS refers to the presence of similar endometrial tissue outside the uterine cavity. It can be broadly divided into three subtypes: superficial EMS, ovarian EMS cysts, and deep infiltrative EMS.^166^ Clinical manifestations include intermittent pelvic pain (dysmenorrhea), chronic acyclic pelvic pain, and infertility.^167^ The pathogenesis of EMS is unclear and mainly includes the theory of menstrual blood reflux implantation theory, the estrogen and progesterone receptor theory, the mechanism of immunomodulation, genetics, and the stem cell theory.^168^ We will not discuss these too much here. We will focus on endometrial repair and regeneration in EMS.

The effects of EMS on cyclic endometrial repair and regeneration include the following: first, hormonal regulation of the shedding of endometrium from abnormal foci may lead to tissue buildup and blockage of the uterine cavity, which ultimately interferes with endometrial regeneration. Second, the shedding and proliferation of ectopic endometrium may lead to rupture and scarring of the surrounding tissues, preventing cyclical endometrial repair and regeneration, and affecting the structure and function of the endometrium. About 10–30% of patients with EMS have IUA, which can cause recurrent bleeding and healing in the uterine cavity when endometrial tissue is shed and grows in the uterus, causing fibrosis of the surrounding tissues and eventually leading to the formation of adhesion bands and causing infertility.^169^ Third, ectopic lesions may cause an inflammatory response and immune system activation, which subsequently prevents endometrium regeneration.

In addition to the diseases mentioned earlier, the remaining diseases include endometrial polyps and endometrial cancer. Clinical treatment modalities include treatment by hormone therapy, antibiotic therapy, radiation therapy, and lesion excision. Combining the current studies and our speculations, stable endometrial repair and itself are strongly associated with the incidence of various diseases. Further, the incidence and progression of infertility in patients depend on the endometrial condition of the woman. Only in the state of ensuring the health and integrity of the patient's endometrium can we talk about improving reproductive outcomes.

### 3.2 Methods/devices to promote endometrial repair after surgery

It is easy to remove diseased tissue for various diseases, such as fibroids and IUA, but it is not easy to maintain endometrial repair and regeneration. Methods available to promote endometrial repair after surgery are IUDs, ISBs, Foley catheters, HA gels, cross‐linked HA gels, estrogens, drugs, growth factors, and amniotic membranes (Figure 2).

Some of these methods have been shown in clinical studies to be effective in maintaining of stable endometrial repair and regeneration after surgery.^170^ Their primary role is to promote the transformation of the uterus into a normal physiological state and increase the rate of endometrial repair. However, they hardly possess biodegradability, low immunogenicity, suitable residence time, and the ability to promote excellent endometrial regeneration. Although IUDs and ISBs can promote endometrial repair, there is no significant difference in reproductive outcomes.^40^, ^171^ Healy et al. published a statistical analysis of randomized controlled clinical trials published from 1989 to 2014, entitled “Intrauterine adhesion prevention after hysteroscopy: a systematic review and meta‐analysis,” which concluded that there is little clear evidence that the current clinical treatment has a significant effect on endometrial repair after hysteroscopic TCRA.^172^, ^173^ While postoperative adjuvant therapy can promote endometrial repair to some extent, we still need to find a variety of effective methods. Rebuilding a normal functioning endometrium is the key to reproduction.^174^ In addition to the strategies mentioned above, stem cell therapy positively affects endometrial regeneration. It can have a better positive effect on tissue injury and fibrotic processes.^175^ The advantages of application in the uterine cavity are the absence of immune rejection and the promotion of endometrial regeneration while reducing endometrial fibrosis. The disadvantages are that stem cells cannot be used directly in tissue wounds, and the survival time of stem cells alone needs to be longer. This result may further inhibit the ability of stem cells to regenerate in the uterine cavity.^176^ In the current state of research, we hope to find a series of scaffolds that promote endometrial repair while maintaining regular cellular activity.

#### 3.2.1 Intrauterine device

The intrauterine device (IUD) is easy to use and can be chosen in a T‐shape, O‐shape, or uterine shape to support the cervix until it fits, according to the degree and location of endometrial injury. Early studies on IUDs showed that IUD placement after TCRA maintained the balance of endometrial regeneration, prevented the recurrence of adhesions, and significantly improved menstrual flow.^177^, ^178^ The disadvantage is that IUDs are primarily made of metal, which can cause an inflammatory immune response when placed in the body. However, the current uterine‐type IUD consists of stainless steel wires that can carry anti‐inflammatory drugs inside, which may better promote endometrial repair, inhibit endometrial fibrosis, and have better prospects for clinical use.^179^ Reed et al.^180^ published a study in the Lancet on the association between IUDs and uterine perforation. The results suggest that we carefully consider using IUDs to avoid the risk of uterine perforation.^180^ Meanwhile, the IUD is insufficient in size and may not cover more significant wounds. Other adjunctive therapies, such as hormonal assistance and gel injection, often accompany the current clinical use of IUDs.^181^ A further problem is that there is no definite type or duration of IUD use in endometrial repair. If implanted in the uterine cavity for long, it can lead to sterile inflammation, infection and bleeding. Eventually, it will still lead to endometrial fibrosis, which cannot be prevented.

#### 3.2.2 Foley catheter

The evolution of the Foley catheter is similar to that of the IUD, initially designed for urinary catheterization. However, as medical research progressed, it was recognized that it could also help repair the endometrium. As a physical barrier, it isolates the traumatic opening and supports the uterus back to its normal size. Compared with the IUD, the soft material of the Foley catheter offers a better risk reduction for complications such as infection. Placement of a Foley catheter after TCRA can maintain the balance of endometrial regeneration, improve the patient's menstrual status and reduce the degree of fibrosis in large traumatic areas.^182^ Regarding the investigation of pregnancy outcomes, Orhue et al.^183^ found more favorable pregnancy outcomes with Foley catheter implantation versus IUD implantation (33.9 vs. 22.5%, *p* < 0.05) and better menstrual recovery (81.4 vs. 62.7%, *p* < 0.05). The Foley catheter was also found to have a more effective endometrial repair than the IUD, while the intrauterine balloon was particularly beneficial in endometrial regeneration.^184^

#### 3.2.3 Intrauterine suitable balloons

In 2014, a heart‐shaped intrauterine balloon suitable for hysteroscopic surgery was proposed (Patent No. CN201420679083.7). The use of balloons in hysteroscopic surgery serves two primary purposes. First, it assists local hemostasis, particularly in surgeries involving submucosal myomectomy procedures, where the entire uterine cavity is invaded, and the endometrial trauma is extensive. This balloon can be used for postoperative hemostasis. Second, it allows better support of both sides of the uterine cavity and the uterine horns, preventing contact with the traumatic surface and reducing the risk of adhesions. The balloon is a drainage device for exudate during the uterine cavity's opening and a delivery channel for internal medications, including antibiotics and anti‐inflammatory drugs.^185^ The advantages are isolation of the wound and drainage of blood or inflammatory exudate, as well as a reduced chance of infection and recurrence. However, there are also disadvantages associated with using balloons, such as the potential for infection and endometrial injury, which could adversely affect wound repair.

Additionally, the placement of the balloon inside the body may cause inconvenience to the patient's daily life. A retrospective study conducted on 150 patients with moderate to severe IUAs revealed that intrauterine balloon (ISB) placement after TCRA was more favorable compared with using a Foley balloon. The ISB showed better outcomes in improving menstrual status and maintaining endometrium repair and regeneration (as measured by the reduction in the American Fertility Society [AFS] score).^186^ Another study by Zhao et al.^187^ suggests combining the Foley balloon with IUD after TCRA may be more effective than using an IUD or Foley balloon alone in endometrial regeneration. However, Lin et al.^121^ found no significant difference between balloons and IUDs in endometrial repair. Therefore, more clinical trials on maintaining endometrium repair and regeneration are needed to find better stabilizing endometrial repair strategies.

#### 3.2.4 Amniotic membrane transplantation

The anti‐infective properties, proendometrial repair function and postplacement safety of traditional physical barriers still require further investigation and validation. The amniotic membrane has made a significant contribution to the field of ophthalmology. Benefits of its use in ophthalmology include reconstruction of the ocular surface structure, maintenance of ocular surface stability, promotion of rapid corneal or conjunctival epithelialization, reduction of scarring, and reduction of inflammatory response.^188^, ^189^ As clinical trials progress, it has been found that amniotic membranes can also prevent postoperative scar formation. The use of amniotic membranes has several advantages over traditional methods. First, the amniotic membrane possesses a variety of differentiated functions. Second, it is easy to apply and adhere to the wound surface for endometrial repair.^190^ Third, promotion of endometrial repair and antifibrosis: the amniotic membrane contains multiple growth factors, such as epidermal growth factor, fibroblast growth factor, and platelet‐derived growth factor, which can better promote endometrial repair and antifibrosis.^191^ Fourth, proproliferative function: the amniotic membrane, as an excellent ECM, can enhance cell adhesion, promote proliferation migration, inhibit apoptosis and promote endometrial repair.^192^ Fifth, low immunogenicity is the most important, and no immunosuppressive therapy is required after reinsertion.^193^, ^194^

Over the past decade, using amniotic membrane as a postoperative graft for hysteroscopic surgery has gained popularity. Although its potential to promote endometrial repair and regeneration was reviewed as early as 2004, its reliability remains to be tested.^195^ Gan et al.^123^ used a catheter to deliver amniotic membrane to the endometrial injury, while the control group was not covered with an amniotic membrane. The results showed that menstrual improvement was significantly better in the amniotic membrane group than in the control group (the score was 31.3 vs. 25.1, *p* < 0.001). However, the pregnancy rates was not significantly different (*p* = 0.237 and *p* = 0.576, respectively).^123^ Another clinical trial in Chongqing, China, aimed to compare the difference between amniotic membrane and chitosan hydrogel in endometrial repair and regeneration. Before making the comparison, we ensured, as much as possible, that there was no significant difference in preoperative endometrial thickness between the two groups (*p* = 0.442). Postoperatively, the amniotic membrane group exhibited significantly better endometrial thickness than the chitosan group (*p* = 0.000). Follow‐up observations of the two groups of patients showed that the newborn abnormal fibrous tissue was significantly lower in the amniotic membrane group than in the chitosan group at 1 and 3 months after surgery (*p*_1_ = 0.000, *p*_2_ = 0.000).^196^ Related research continues, Amer et al.^197^ studied the temporary placement of an amniotic barrier after hysteroscopic surgery. The results demonstrated that the amniotic membrane reduced the regenerative abnormal fibrous tissue and promoted endometrial repair (*p* = 0.003 and *p* = 0.01).^197^ Zheng et al.^198^ performed an extraction analysis of the amniotic membrane placement (case number: 300). They found that placement of the amniotic membrane after hysteroscopic surgery significantly improved patients' menstrual status (*p* < 0.001). However, there were no significant changes in the rates of fibrous tissue recurrence (*p* = 0.290), pregnancy (*p* = 0.260), and spontaneous abortion (*p* = 0.750) were not significantly changed.^198^

As previously mentioned, the multiple differentiation functions of amniotic cells are also a significant advantage. Having reached their conclusions, Amer et al.^199^ conducted a study investigating the mechanism behind the amniotic membrane's promotion of endometrial repair and reduction in recurrence rates. After performing amniotic membrane transplantation in a patient who had undergone TCRA and conducting long‐term investigations, they concluded that it could be a significant source of stem cells for endometrial repair, as confirmed with CD10 immunohistochemistry.^199^ This research has led to an increasing number of individuals combining amniotic and regenerative medicine. Gan et al. extracted human amniotic mesenchymal stromal cells (hAMSCs) and used them for transplantation to promote endometrial growth. The findings revealed significant differences, including a thicker endometrium, a denser number of glands and fewer areas of fibrosis. Immunological analysis revealed that transplantation of hAMSCs decreased proinflammatory cytokines while increasing anti‐inflammatory cytokines compared with the injured uterine horns.^200^ It is worth noting that not only is an amniotic membrane available for maintaining tissue homeostasis, but small intestinal submucosa (SIS),^201^ platelet‐rich plasma (PRP),^202^ and urinary bladder matrix (UBM)^203^ are also still available to promote endometrial repair and regeneration.

#### 3.2.5 Clinical medications used for cyclical endometrial repair and regeneration

Our goal is to “maintain cyclical endometrial repair and regeneration.” With the growing utilization of clinical drugs, many small molecules have been discovered to affect endometrial regeneration positively (Table 2).

**TABLE 2:** Pharmacotherapy in cyclical endometrial repair and regeneration.

Method	Specific type	Drug administration route	Research type	Advantages	Adverse drug reactions	References
Hormone	Estrogen	Oral administration	Clinical research	Promote endometrial regeneration, dissolve and cover scar tissue	Nausea, vomiting, thrombosis	^206^
		Oral administration	Clinical research	(4 mg) Inhibit fibrosis and improve the receptive capacity of endometrium	Nausea, vomiting, headache, dizziness, thrombosis	^211^
		Oral administration	Clinical research	(9 mg) Promote the proliferation and differentiation of intimal cells and inhibit fibrosis	Nausea, vomiting, headache	^212^
		Oral administration	Clinical research	(4 mg) Promote endometrial regeneration, lower adhesion, better intimal recovery, better AFS score	Nausea, vomiting, headache	^213^
		Oral administration	Clinical research	(2 mg) Improvement of menstruation, reduction of adhesion	Nausea, swelling, thrombosis, abnormal liver function	^214^
	Growth hormone	Oral administration	Clinical research	Promote proliferation and vascularization, upregulate molecular expression, promote intimal regeneration	Hyperglycemia	^215^
		Oral administration	Clinical research	Synergistic with estrogen to promote intimal regeneration and improve estrogen utilization	Hyperglycemia	^216^
Drugs	Aspirin	Intrauterine balloon delivery	Clinical research	Promote intimal hyperplasia, improve adhesion, optimize menstrual state, prevent thrombus	Nausea; vomiting; allergy; hepatotoxicity	^217^
		Oral administration	Clinical research	Suppressing the TGF‑β1–Smad2/Smad3 pathway, promote angiogenesis, improve menstrual score	Nausea; vomiting; indigestion	^218^
		Oral administration	Clinical research	Promote intimal regeneration, promote angiogenesis	Gastrointestinal reaction	^219^
	Metformin	Intraperitoneal injections	Rat research	Increase the number of glands, reduce fibrotic areas, promote vascularization	Abdominal pain, diarrhea, nausea and vomiting	^220^
	Sildenafil	Hydrogel sponge transportation	Clinical research	Promote intimal hyperplasia, increase blood perfusion, promote angiogenesis	Unknow	^221^
		Oral administration	Clinical research	Increase uterine blood flow, improves ovulation success rate	Unknow	^222^
	Exenatide	injected subcutaneously	Rat research	Reduce collagen deposition, promote intimal gland proliferation	Unknow	^223^
	Prunella vulgaris oil	Oral administration	Rat research	Anti‐inflammation, antifibrosis, promoting intimal regeneration	Unknow	^224^
	Traditional Chinese herb	Oral administration	Clinical research	Promotes blood circulation, removes blood‐stasis	Unknow	^225^
	Mitomycin C	Oral administration	Rat research	Promote fibroblast apoptosis, inhibits endometrial fibrosis	Unknow	^226^
Physical method	Acupuncture	External Use	Clinical research	Promote uterine blood circulation	Unknow	^227^

Endometrial shedding results from the synergistic interaction of several hormones, represented by estrogen and progesterone.^204^ Most postoperative adjuvant hormone therapies also involve the use of these hormones. Estrogen is widely recognized for its ability to promote endometrial regeneration in gynecological diseases, as well as accelerate wound healing and vascular regeneration.^205^ In reality, hormonal adjuvant therapy after hysteroscopic surgery is essential. Studies have demonstrated that estrogen therapy after hysteroscopic surgery can promote endometrial repair, improve menstrual status, and reduce the risk of adhesions to a certain extent.^206^ Dreisler et al.^207^ demonstrated through numerous nonclinical and clinical trials that estrogen reduces fibrous tissue growth and improves endometrial regeneration and pregnancy outcomes. More reliable studies sufficiently confirm the importance of estrogen in promoting endometrial repair and regeneration. However, clinical problems arise, as there is no strict international standard for estrogen dosage, route of administration, duration of therapy and safety. Overdosing on estrogen can lead to excessive endometrial hyperplasia and irregular blood contact. The AAGL Practice Guideline and the European Society of Gynecological Endoscopy recommend hormone therapy in guidelines on IUAs. However, there is still controversy regarding the dosage and periodicity of use.^40^, ^208^ The most common route of administration is oral estrogen, but its effectiveness is reduced as the liver metabolizes it after oral intake (only 1–3% remains active). Limited studies on transuterine or dermal estrogen supplementation are still needed, and long‐term follow‐up studies are still needed. Current cutting‐edge research focuses on using biocompatible and biodegradable hydrogels/scaffolds to transport estrogens to the endometrium,^209^ improving utilization while reducing side effects.

Another standard treatment modality is growth factor/cytokine adjuvant therapy. For example, VEGF and bFGF are essential for endometrial stromal cell proliferation, neovascularization and glandular differentiation.^210^ When the endometrium is injured, and growth factors/cytokines cannot be synthesized or are functionally blocked, it becomes apparent that we can use carriers to deliver growth factors/cytokines to the inside of the uterus in the same way as stem cells. A more detailed section on delivery strategies will later be mentioned. Other therapeutic modalities included aspirin and sildenafil (Table 2). Undoubtedly, the above treatment strategies are of great significance for endometrial regeneration. However, there are currently no apparent limits regarding the dosage, route of administration, and treatment duration of drug therapy. Further relevant clinical trials are still required in the future to ensure safety.

#### 3.2.6 Hydrogels used clinically to maintain endometrial repair

HA, known for its high biocompatibility and natural properties, serves as a crucial barrier in promoting endometrial repair and inhibiting excessive fibrosis.^228^, ^229^ The clinical trade name is “Ankang Gong” (related properties and cutting‐edge research will be described later).^230^ In early clinical trials of HA alone, SeiedeZahra GhanadzadehTafti found that HA can reduce area of fibrotic tissue after hysteroscopic surgery.^231^ Several prospective studies have shown that HA reduces adhesions compared with IUDs or balloons. Endometrial regeneration and menstruation were also better in patients with postoperative HA implants during long‐term follow‐up.^232^ However, many studies have confirmed that simple physical barriers or drugs are ineffective in promoting endometrial repair and maintaining tissue homeostasis. After 5 years of follow‐up, Thubert et al.^233^ found that HA could not restore the abnormally shed endometrium to a normal functional condition and could not interfere with the outcome of pregnancy. Consequently, an increasing number of biomedical scientists have been exploring the transport of antiadhesion substances through HA to enhance their regenerative capabilities, which holds significant implications for the strategy of endometrial repair.^234^ Improving the transport system can better promote intimal regeneration and inhibit the process of fibrosis.

In pursuit of expediting endometrial regeneration, poorly biodegradable devices have been used to isolate the adhesions at the traumatic wound. Extensive research and long‐term follow‐up investigations have revealed the potential of drugs that stimulate endometrial regeneration and biodegradable biomaterials in facilitating endometrial repair and regeneration. Ongoing development and exploration have led to the emergence of biomaterials with outstanding performance, which are progressively becoming the prevailing treatment choices.

## 4 THE FUTURE OF REGENERATIVE MEDICINE IN CYCLICAL ENDOMETRIAL REPAIR AND REGENERATION

The fields of biomedical engineering and tissue engineering within regenerative medicine have experienced substantial growth in recent years. Notably, advancements in clinical medicine and material preparation technology have driven the creation of diverse biomaterials featuring physical barriers, biodegradability, and exceptional histocompatibility. These attributes render them ideal candidates for carrying drugs, active substances, and stem cells.^235^ Biomedical materials have gained increasing significance in clinical diagnosis and treatment. Historically, the origins of biomedical materials can be traced back to 2500 BC, as evidenced by artifacts like hemostatic cotton and artificial dentures.^236^ Biomedical materials typically encompass natural or synthetic substances, capable of use independently or in conjunction with other materials, drugs, or active substances for tissue repair and the treatment of organ injuries. Crucially, they do not pose a risk of harm to the human body during utilization. Typical applications of biomedical materials encompass bone scaffolds,^237^, ^238^ cardiovascular scaffolds,^239^, ^240^ skin excipients,^241^ drug delivery,^242^, ^243^ among others.

**FIGURE 3:**
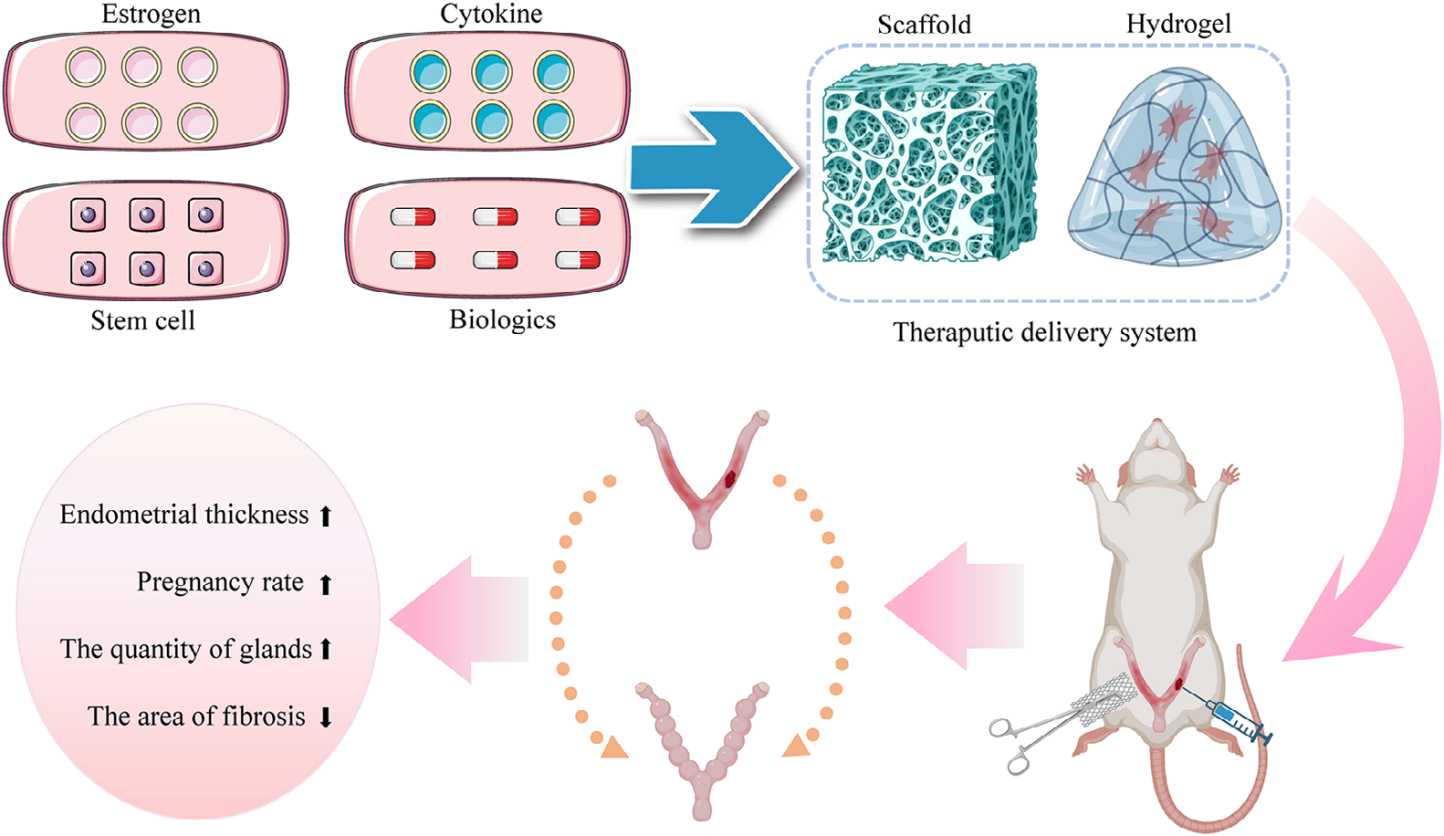
Scaffolds/hydrogels encapsulate therapeutics such as estrogen, cytokine, stem cells, or biologics to perform endometrial repair in rats.

Interventions for cyclical endometrial repair and regeneration use biomaterials that can accelerate cell migration, glandular production, and blood vessel regeneration, and maintain the stability of the repair through the properties of the material or the therapeutic effects of the drug.^244^ Future goals should encompass the restoration of normal uterine function and the enhancement of implantation and pregnancy rates, taking into account factors such as embryo size, quantity, and quality. Specifically, the materials employed should possess attributes like biodegradability, excellent biocompatibility, good mechanical properties, noninflammatory, nonimmunogenic characteristics, and sustainability.^245^, ^246^, ^247^ Considerations should extend to restoring organ intimal repair function, enhancing reproductive capacity, and facilitating the carriage of drugs, active substances, or stem cells (Figure 3). Importantly, the prepared composites require no sutures to sustain activity, remain functional in the presence of blood, and are user‐friendly. Superior composite scaffolds exhibit properties that include inflammation mitigation, fibrosis reduction (attenuating fibrin signal while enhancing fiber inhibition signal), and inhibition of fibrin tissue formation.^248^, ^249^ Biomedical materials frequently employed in endometrial repair fall into three categories: hydrogels, scaffolds, and films (Table 3).

**TABLE 3:** Biomaterials used as scaffolds or hydrogels for endometrial repair and regeneration.

Biomaterial	Active substance	Biomaterial‐based features	Advantages	Model (method; experimental period); experimental group	Mechanism	References
Hyaluronic acid hydrogel	/	Degradable, nonbiotoxic, inhibits inflammation, improves epithelialization	Significantly promoted endometrial regeneration (7.5 mm > 6.5 mm > 5 mm for IUA + HA groups, HA groups, IUD groups, respectively)	Clinical trial (12 weeks; phase I clinical trial); IUD; IUA + HA:HA (34:34:34)	/	^39^
	Estradiol	Excellent histocompatibility, dynamic viscosity	Mild improvement of adhesions, reduced recurrence rate and improved menstruation, promotes endometrial repair	Clinical trial (8 weeks; phase I clinical trial); the intervention group versus the control group (84:87)	/	^185^
	/	Excellent biocompatibility, degradability, nonimmunogenicity	Significant reduction in adhesion rate (14% in HA vs. 32% in control) and improvement in adhesion score (*p* = 0.012)	Clinical trial (*n* = 34; 2 months; phase II clinical trial); the case group (*n* = 34) and the control group (*n* = 31)	/	^231^
	/	/	Significantly improved IUA prevalence (13.0 vs. 30.6%); reduced patient adhesions	Clinical trial (12 months; phase II clinical trial); the intervention group versus the control group (77:72)	/	^250^
	/	/	Women in the intervention group had significantly fewer pregnancies (77.6 vs. 92.2%; *p* < 0.05), lower miscarriage rates (20.9 vs. 37.5%; *p* < 0.05), significantly more ongoing pregnancies (94.3 vs. 71.7%; *p* < 0.01); and improved menstrual status and bleeding	Clinical trial (42 months; phase II clinical trial); the intervention group versus the control group (67:64)	/	^251^
	/	/	Reduced incidence of adhesions (9.5 vs. 24.1%; *p* < 0.05); significantly lower adhesion and menstrual patterns and cumulative adhesion scores (*p* = 0.0007, .008, 0.0012, and 0.0006, respectively); and significantly fewer patients with moderate to severe IUA (*p* < .05)	Clinical trial (3 months; phase II clinical trial); the intervention group versus the control group (150:150)	/	^252^
	/	Bioabsorbable, sterile, transparent, high viscosity	Significantly reduced adhesion rate, adhesion score (*p* < 0.05)	Clinical trial (3 months; phase I clinical trial); *n* = 85	/	^253^
	/	Absorbable, good biocompatibility, maintains healing tissue separation	Reduced adhesion recurrence rate (0 vs. 21.43%; *p* = 0.07)	Clinical trial (6 months; phase I clinical trial); the intervention group versus the control group (30:30)	/	^254^
	/	Degradability, excellent biocompatibility	Reduced IUA recurrence rate (12.8 vs. 39.1%, *p* = 0.012); reduced degree of adhesions (*p* = 0.002)	Clinical trial (12 weeks; phase II clinical trial); the intervention group versus the control group (35:35)	/	^255^
	/	Biodegradable and noninflammatory	Reduced recurrence of IUA (31.1 vs. 39.8%); mild improvement in menstrual status (87.7 vs. 76.4%)	Clinical trial (3 months; phase II clinical trial); the intervention group versus the control group (122:123)	/	^256^
	/	Biodegradable and noncytotoxic	Significantly lower incidence of adhesions (13.95 vs. 31.70%; *p* < 0.05); significantly lower adhesion scores (*p* < 0.05)	Clinical trial (3 months; phase II clinical trial); the intervention group versus the control group (46:46)	/	^257^
	/	Degradability, excellent biocompatibility	Significant reduction in adhesion recurrence rate (10.44 vs. 26.15%; *p* < 0.05); reduction in the degree of adhesion (*p* < 0.05)	Clinical trial (3 months; phase II clinical trial); the intervention group versus the control group (66:66)	/	^258^
	/	Transparent, good security	Significantly increased fetal number (3.7 > 2.1; *p* < 0.05); increased fetal survival (3.4 > 1.9, *p* = 0.05)	New Zealand White Rabbits (curettage; 21 days); the intervention group versus the control group (10:10)	/	^259^
	Mesenchymal stem cells (MSCs)	Porous, degradable, excellent biocompatibility, continuous cell release (complete release within one week), can support cell proliferation	Promotion of epithelial and endometrial cell proliferation, thicker endometrium and higher number of glands in the treated group (treated side: control side = 5:1, *p* < 0.01)	Rat Model (electrocoagulation;14 days); the intervention group versus the control group	Stimulation of paracrine signaling pathways to promote endometrial regeneration and increase levels of growth factors (EGF, IGF, FGF, etc.)	^260^
	Apoptotic bodies (ABs)	Good biocompatibility, functionality, biodegradability, injectable, encapsulate Abs (entrapment efficiency is 89.2 ± 0.9%), sustained release Abs (5 day—95%)	Promote endometrial cell proliferation (17,115 ± 565.6 μm > 12,146 ± 392.5 μm); thicker endometrium and more glands in HA/Abs group. Pregnancy rates were significantly higher in the HA/Abs group than in the NR and ABs groups; better vascular regeneration in HA/Abs group	Rat Model (reperfusion injury of uterus; 60 days); NR group (natural repair without any treatment); Abs group (injection of the AB solution only); HA group (injection of HA only); HA/Abs group (injection of the AB‐laden HA hydrogel)	Activated mitochondrial‐enzymes; inhibited IL‐1β, TNFα, IL‐6, IFN‐γ activation, enhanced VEGFA, IGF1, IL‐10, VEGFA, IGF1, TGFβ expression.	^261^
	The human placenta‐derived mesenchymal stem cells (HP–MSCs)	Biodegradability, excellent carrying capacity, sustainable drug release, porosity, suitable elasticity, and viscosity	Endometrial thickness was much greater than in the ethanol group (292.3 ± 19.14 μm > 171.3 ± 14.59 μm); more glands; pregnancy success rate was much higher than in the ethanol group (5.889 ± 0.539 > 3.273 ± 0.506); better vascular regeneration; less fibrous tissue than in the ethanol group (0.279 ± 0.016 < 0.477 ± 0.027)	Rat Model (vaginal injection of ethanol; 2 weeks); ethanol group, PBS group, HA hydrogel group, HP–MSCs group, HP–MSCs–HA group	Promotes VEGF expression; activates JNK/Erk1/2–Stat3–VEGF pathway; activates Jak2–Stat5 and c‐Fos–VEGF path way	^262^
	Human umbilical cord‐derived mesenchymal stem cells (huMSCs)	Excellent biocompatibility, noncytotoxic, biodegradable	Promoted glandular hyperplasia (4.9662 ± 1.4935, *p* < 0.01); reduced fibrotic area (5.5955 ± 3.6572%%, *p* < 0.01); promoted endometrial repair (4.2667 ± 0.55 mm, *p* < 0.01)	Rhesus monkeys Model (mechanical injury; 2 months); HA‐GEL transplantation group; huMSCs /HA‐GEL transplantation group	Increased IGF‐Q‐1, EGF, BDNF, IL‐4 expression (*p* < 0.05); inhibited IFN‐γ (*p* < 0.01)	^263^
	Adipose‐derived mesenchymal stem cells (ASCs)	Injectability, degradability	Fibrosis was lower than in the model group (7th day *p* = 0.049; 10th day *p* = 0.026; 14th day *p* = 0.004; 21st *p* = 0.008); promoted epithelialization, reduced adhesion band formation	Rat Model (scrape the endometrial lining; 21 days); the IUA model group, gel therapy group, and combination therapy group	Inhibited Smad3 (*p* < 0.05); promoted LIF expression (on days 10 and 14 (*p* < 0.05)	^264^
Carboxymethylcellulose and hyaluronic acid (CH) hydrogel	/	Excellent biocompatibility, water capacity, and viscoelasticity	Inhibits inflammation, promotes tissue granulation, and re‐epithelialization	Clinical trial (n = 95; 4 weeks; phase II clinical trial); *n* = 95	/	^265^
Alginate, carboxymethylcellulose, and hyaluronic acid (ACH) hydrogel	/	Excellent biocompatibility, hemostasis, antibacterial	Promotes vascular regeneration, inhibits inflammation, and regulates dysfunctional uterine bleeding	Clinical trial (*n* = 92; 4 weeks; phase II clinical trial); *n* = 92	/	^265^
Hyaluronic acid/fibrin hydrogel	Decidualized endometrial stromal cells (EMSCs)	Porosity, good biocompatibility, degradability, support cell migration growth, promote stem cell differentiation	HA/fibrin hydrogels group had thicker intima (*p* < 0.01), more viable tissue and less fibrous tissue; improved embryo implantation rate	Rat Model (vaginal injection of ethanol; 2 weeks); the intervention group versus the control group	Cadherin‐1 and fibronectin levels were upregulated (*p* < 0.01); IGF‐1 and ER levels were upregulated; PECAM and IGF‐1 levels were higher (*p* < 0.01)	^266^
Aloe/poloxamer hydrogel	β‐Estradiol, the decellularized uterus‐derived nanoparticles (uECMNPs)	Excellent biocompatibility, biomimetic sensitive property (4°C‐37°C), noncytotoxic, low immunogenicity, sustained release of E2 (E2@uECMNPs/AP‐51. sensitive property (4°C‐37°C), noncytotoxic, low immunogenicity, sustained release of E2 (E2@uECMNPs/AP‐51.81 ± 5.11%; E2/AP‐90 ± 4.31%)	The best promotion of cell proliferation and wound healing (34.19 ± 1.87% at 24 h; 44.37 ± 1.76% at 48 h); fibrosis area was much smaller than in the IUA group (51.09 ± 2.63% < 74.74 ± 4.36%, <0.05); best condition of gland number and endometrial thickness (498.33 ± 26.26 μm, 21 ± 2 per HPF)	Rat Model (scrape the endometrial lining; 7days); sham group, IUA group, E2 alone group, commercially avail able E2 gels group, AP group and E2@uECMNPs/AP group	Enhance cytokeratin (CK), Ki67 expression; inhibit TNF‐ɑ, TGF‐β1 expression	^267^
Heparin–poloxamer (HP) hydrogel	17β‐Estradiol (E2)	Temperature sensitive property (37°C), nontoxic, excellent biocompatibility, three‐dimensional structure similar to the porous sponge	Promotes endometrial regeneration (two‐dimensional ultrasound); increases the number of glands, decreases the fibrosis	Rat Model (scrape the endometrial lining; 14 days); sham operation group, IUA group, HP hydrogel groups, E2–HP hydrogel group	Activation of PI3K/Akt and ERK1/2, inhibition of endoplasmic reticulum stress signaling, upregulation of VEGF levels, and reduction of TGF‐β levels	^268^
Heparin‐ε‐polylysine (EPL)–poloxamer hydrogel	Keratinocyte growth factor (KGF)	Temperature sensitive property(37°C), bioadhesivity, biodegradable properties, porous mesh‐like structures, continuous release of KGF (7 days—80%)	Restoration of basal layer tissue morphology, promotion of submucosal and basal layer glandular regeneration, increased endometrial regeneration, provascularization	Rat Model (scrape the endometrial lining; 7 days); Model Group, EPL–HP group, KGF–HP hydrogel, and KGF–EPL–HP hydrogel	Promotion of neointima‐related cytokeratin (CK) and ki67 expression	^269^
Hydrogel pluronic F‐127 (PF‐127)	Bone marrow stromal cells (BMSCs), Vitamin C (Vc)	Low toxicity, biocompatibility, thermo‐reversibility	Improves cell survival, reduces fibrosis area, and promotes endometrial repair	Rat Model (scrape the endometrial lining; 2 weeks); A sham group, an IUA control group, an IUA BMSC encapsulated in PF‐127 plus Vc group, an IUA BMSC plus Vc group and an IUA PF‐127 plus Vc group	Promotes IL‐10 secretion and reduces IL‐6 and TNF‐α levels	^270^
Heparin‐modified poloxamer (HP) hydrogel	Keratinocyte growth factor (KGF)	Temperature sensitive property, biodegradable, suitable mechanical properties, multivacancy mesh structure, sustainable release of KGF	Reduced fibrosis area, reduced collagen fibers, significantly increased number of glands, denser endometrial epithelial cells (EEC) microvilli, promoted angiogenesis, increased pregnancy rate (66.6% in KGF–HP hydrogel group and 11.1% in IUA group, respectively)	Rat Model (scrape the endometrial lining; 90 days); sham group, IUA group, group treated with HP hydrogel, group treated with E2 (2.5 mg/mL, Estradiol benzoate), group treated with KGF (2.5 mg/mL), group treated with KGF–HP hydrogel (equivalence of 2.5 mg/mL)	Promote LC3‐II expression and downregulate P62 level	^271^
Heparin–poloxamer (HP) hydrogel	17β‐Estradiol (E2)	Nontoxic, biodegradable, temperature sensitive property (37°C), three‐dimensional structure resembling a porous sponge	Isolation of traumatic wounds, promotion of epithelial cell regeneration, inhibition of apoptosis, promotion of glandular regeneration, improvement of pregnancy rate	Rat Model (scrape the endometrial lining; 32 days); IUA group, HP hydrogel group, E2 solution group and E2–HP hydrogel group.	Upregulation of proliferating cell nuclear antigen (PCNA) and bFGF levels; reduction of caspase‐3 and Bax levels, activation of MAPKs p38 and ERK1/2 pathways	^272^
Chitosan–heparin hydrogel	Stromal cell‐derived factor‐1α (SDF‐1α)	Temperature sensitive property (37°C), biocompatibility, biodegradability, nontoxicity, antibacterial activity, suitable drug half‐life (24 h—18%; 120 h—53%), porosity	Promotes cell adhesion, reduces fibrosis, promotes endometrial repair, promotes glandular proliferation and promotes vascular regeneration	Rat Model (scrape the endometrial lining; 7 days); Group A (Control), group B (no treatment), group C (treated with hydrogel only), group D (treated with SDF‐1α hydrogel (total 2 μg in 0.1 mL hydrogel each uterine horn), group E (treated with SDF‐1α (2 μg in 0.1 mL normal saline each uterine horn)	Upregulation of TGF‐1 and VEGF levels	^273^
Poly(hydroxyethyl methacrylate) (PHEMA) hydrogel	17β‐Estradiol (E2)–SiO₂ (E2@SiO₂)	Good biocompatibility, good mechanical properties, porous, sustained release of E2 (46.02 ng d^−1^)	Promotes endometrial cell proliferation and inhibits fibrosis	Rat Model (scrape the endometrial lining), sham group, IUAs group, PHM group, PHM‐Si group	/	^274^
FGF1 silk sericin hydrogel material (FGF1‐SS hydrogel)	FGF1 factor	Excellent biocompatibility, nontoxic, release FGF1 stably for a long time	Significantly promoted the migration and infiltration capacity of ESCs, restored the gland number and uterine wall thickness, inhibited fibrosis and increased pregnancy rate in rats (65.1 ± 6.4%)	Rat Model (injecting 95% ethanol to injure the uterus; 60 day); normal group (control), sham group (sham), model group (model), WT treatment group (WT) and WTþFGF1 treatment group (WT + FGF1)	Activation of the TGF‐b/Smad pathway, the	^275^
Poly(polyethylene glycol citrate‐co‐*N*‐isopropylacrylamide) (PPCN)–gelatin hydrogel	Human amniotic mesenchymal stem cells (hAMSCs)	Thermoresponsive (37°C), noninflammatory, noncytotoxic, well biocompatible	Promotes cell proliferation and differentiation, enhances endometrial thickness, increases the number of endometrial glands, reduces the abrasion area and increases pregnancy rate	Rat Model (injecting 95% ethanol to injure the uterus; 60 day); the sham‐operated group, the IUA group, the PPCNg group, the hAMSCs group, and the PPCNg/hAMSCs group (*n* = 16 in each group)	Upregulation of CK7, CK19, Ki67, VEGF, ER, PR levels	^276^
Poly(ethylene glycol)‑b‑poly(l‑phenylalanine)/poly(ethylene glycol) or cross‐linked hyaluronic acid hydrogel (CLHA)	/	Excellent biocompatibility, noncytotoxic	Promotes endometrial repair, accelerates glandular regeneration, significantly reduces fibrosis, and increases pregnancy rate	Rat Model (scrape the endometrial lining; 12 day + 1 week); sham‐operated group (OS), model group, CLHA group	Inhibition of TGF‐β1 expression and regulation of Muc‐4 promote embryo fertilization	^277^
Collagen scaffolds	Basic fibroblast growth factor (bFGF)	Excellent biocompatibility, noncytotoxic, low immunogenicity	Promoted the endometrial angiogenesis, increase endometrial thickness	Clinical trial (phase I clinical trial)	/	^278^
	Vascular endothelial growth factor (VEGF)	Excellent biocompatibility, noncytotoxic, low immunogenicity	Accelerated gland regeneration, promoted muscle layer regeneration (mean 24.12 ± 3.82 vs. 17.82 ± 2.92 and 16.63 ± 3.54, respectively, *p* < 0.01); promoted vascular regeneration (vascular density: 22.34 ± 4.76 > 16.46 ± 1.76 > 11.15 ± 2.89) and improve the uterine receptivity	Rat Model (resect uterine horn; 90 days); CBD–VEGF groups, native‐VEGF groups and PBS groups	Regulation of a‐SMA levels	^279^
	Basic fibroblast growth factor (bFGF)	Excellent biocompatibility, biodegradability	Promotes vascular regeneration, improves cellularization, promotes muscle layer regeneration, promotes endometrial repair, and inhibits fibrosis	Rat Model (resect uterine horn; 90 days); Collagen/CBD–bFGF groups, collagen/native–bFGF groups, collagen/PBS groups, spontaneous regeneration groups, sham operated groups	Regulation of a‐SMA levels	^280^
	Umbilical cord‐derived mesenchymal stromal cells (UC‐MSCs)	Degradable, excellent biocompatibility	Significantly enhanced endometrial proliferation and differentiation (increased from 4.46 ± 0.85 to 5.74 ± 1.20 mm (*p* < 0.01), promote vascular regeneration	Clinical trial (phase I clinical trial)	Upregulated ERα, Ki67, and vWF expression levels;	^281^
	Human endometrial perivascular cells (En‐PSCs)	Noninflammatory, noncytotoxic, good biocompatibility	Promote regeneration of endometrium and myometrium of injured rat uterus, induce neovascularization, and increase pregnancy rate	Rat Model (resect uterine horn; days + 2 weeks); a sham‐operated group, collagen/PBS group, collagen/En‐PSCs group	/	^282^
	Menstrual blood mesenchymal stem cell (MBMSC)	/	Promoted endometrial cell regeneration, promoted glandular regeneration (*p* < 0.05 vs. M+MBMSCs group), reduced fibrosis (*p* < 0.05 vs. M+MBMSCs group)	Rat Model (scrape the endometrial lining; 90 days); Model group (M), M+CS group, M+MBMSCs group, M+MBMSCs+CS group	Regulate CK18 levels	^283^
	Human umbilical cord‐derived mesenchymal stem cells (UC‐MSCs)	Noninflammatory, noncytotoxic and biocompatible	Maintenance of normal uterine cavity structure, promotion of endometrial regeneration (610 ± 30 mm >220 ± 20 mm > 170 ± 20 mm, compared with groups S and NR), promotion of collagen remodeling (*p* < 0.01, *p* < 0.01, compared with groups S and NR), induction of endometrial cell proliferation, and improvement of fertility outcomes	Rat Model (scrape the endometrial lining; 60 days); the sham group, the natural repair group (NR), the CS group, the CS/UC‐MSCs group	Enhance the expression of estrogen receptor a and progesterone receptor; upregulation of VEGF‐A and TGF‐b1 levels	^284^
	Umbilical cord‐derived mesenchymal stem cells (UC‐MSCs)	Biodegradable properties, low immunogenicity, nontoxic	Increased pregnancy rate (*p* < 0.017, compared with the other three groups), promotion of collagen degradation and promoted regeneration of the endometrium, myometrium and blood vessels in uterine scars	Rat Model (excise uterine horn; 60 days); PBS group, scaffold group, UC‐MSCs group, scaffold/UC‐MSCs group	Upregulates MMP‐9 levels and promotes secretion of paracrine factors (such as FGF2 and VEGF)	^285^
	Bone marrow‐ derived mesenchymal stem cells (BM‐MSCs)	Abundance, biodegradability, biocompatibility	Improved vascular distribution, promoted stem cell proliferation and differentiation, promoted endometrial regeneration (*p* < 0.01, compared with other groups), promoted neovascularization and reconstructed uterine morphology (support the implantation of embryos and development of fetuses)	Rat Model (excise uterine horn; 90 days); a sham operated group, a spontaneous repair group, a collagen/PBS group, a collagen/BM‐MSCs group	Upregulate bFGF, IGF‐1, TGFb‐1, VEGF levels	^286^
	Mesenchymal stem cell‐derived exosomes	Good cytocompatibility, low immunogenicity	Endometrium regeneration, collagen remodeling	Rat Model (scrape the endometrial lining; 60 days); natural repair group (NR), CS group, exosomes infiltration group (Exos), CS/Exos transplantation group (CS/Exos)	Enhanced ERα/PR expressions, increased the expression of the estrogen receptor α/progesteroe receptor	^287^
	Human umbilical cord mesenchymal stem cells (hUCMSC)	Inherent biocompatibility, low antigenicity and biodegradability	Increased the number of endometrial glands, reduced the area of fibrosis (compared with either the collagen scaffold or hUCMSCs alone)	Rat Model (scrape the endometrial lining); sham group, model group, hUCMSCs group, estrogen group, hUCMSCs + scaffold group, estrogen + scaffold group	Upregulate CXCR4 and p‐TAZ protein levels, reduced ER, CTGF, TGFβ1, and FGF2 expression, promoted CK and KI67 expression, accelerated the homing of stem cells	^288^
	Human embryonic stem cells (hESCs)	Biodegradable properties, low immunogenicity, excellent biocompatibility	Promote angiogenesis, recruit cells to the injured site to accelerate natural healing, reduce scar formation	Rat Model (excise uterine horn;12 weeks + 19–22 days); collage group, cells/collagen group, natural regeneration group, sham‐ operated group	Promotes the expression of Hoxa10, Intergrin β3 and LIF and the transcription of genes related to endometrial development	^289^
Polylactides (PLA)–poly (ethylene oxide) (PEO) film	/	Degradation, swelling/deployment, antiadhesion proper tie, excellent biocompatibility	Limiting the adhesion of fibroblasts, reduce the incidence of adhesions	Clinical trial (12 days; phase I clinical trial); the intervention group versus the control group	/	^290^
Poly(d,l‐lactide) (PLA)–poly (ethylene oxide) (PEO) film	/	Nonadhesion properties, biodegradable properties, excellent biocompatibility	Isolation of traumatic surfaces and reduction of adhesion recurrence	Clinical trial (6 weeks; phase I clinical trial); the intervention group versus the control group	/	^291^
Polylactides (PLA)–poly (ethylene oxide) (PEO) film	/	Degradable, excellent biocompatibility, no long‐term tissue irritation, no negative impact on fertility	Reduced adhesion recurrence rate (27, 80, and 100%, respectively)	Rat Model (scrape the endometrial lining; 28 days); DPF group, hyaluronic acid (HA) gel group, sham group	/	^292^

(Concerning the categorization of clinical trial phases in the table, we focused on the trial's duration, the number of participants, and its purpose. The original text seldom provides clear phases for the mentioned trials. This is because they require a clear indicator of the phases. Hence, the clinical trial phases indicated in the table are solely for reference purposes.).

### 4.1 Biomedical hydrogels in cyclical endometrial repair and regeneration

#### 4.1.1 Requirements of biomedical hydrogels

Hydrogels are polymer networks characterized by a three‐dimensional mesh structure capable of absorbing significant amounts of water or tissue fluids.^293^, ^294^, ^295^, ^296^ Hydrogels are regarded as the most promising alternative to the ECM. Not only is their structure very similar to the ECM's, but they can be continuously modified and processed to form a high‐performance artificial ECM during continuous modification and processing.^297^ The hydrogel exhibits specific swelling properties due to hydrophilic groups, such as carboxyl and hydroxyl. Researches have demonstrated that hydrogels can absorb water thousands of times their dry weight while maintaining their structural integrity, indicating their exceptional capacity for drug loading.^298^ At the same time, it is biodegradability, a fundamental requirement for tissue engineering.^299^ It also has good biocompatibility, which prevents it from causing autoimmune reactions. These properties can support hydrogels as excellent carriers for inducing cell proliferation or differentiation and internal environment homeostasis in endometrial injury.^300^, ^301^, ^302^ Hydrogels have recently found extensive application in bone repair,^303^ cardiovascular scaffolds,^304^ vascular regeneration,^305^, ^306^, ^307^ and skin excipients.^308^ The exceptional properties of hydrogels enable them to serve as carriers for estrogens, active substances, drugs, and stem cells, facilitating their transportation into the uterine cavity. Due to its unique three‐dimensional structure, the hydrogel can isolate the traumatic surface. As a carrier, they can effectively promote endometrial repair and restore normal physiological function.^309^, ^310^

The utilization of intrauterine hydrogels hinges predominantly on their characteristics such as gelation time, viscosity, biocompatibility, and degradability. When employing hydrogels in the uterine cavity, it is imperative to account for the varying physiological characteristics of different patients. The uterus undergoes periodic blood drainage, and endometrial peristalsis induces intracavitary fluid movement, furnishing essential nutritional support for embryo implantation.^311^ First, the hydrogel's gelation time should be brief, enabling rapid gelation shortly after injection and improved adhesion to the affected area. Nonetheless, careful consideration is required to maintain a balance between its mechanical and prorepair properties.^312^, ^313^ Second, the hydrogel precursor should exhibit low viscosity facilitating ease of transport into the uterine cavity.^314^ Additionally, it must exhibit excellent biocompatibility to enhance drug or cellular functionality. The hydrogel should undergo gradual in vivo degradation and be eliminable from the uterus through fluid processes.

#### 4.1.2 Hyaluronic acid

HA is a prominent example of natural polymers in biomedical materials. HA serves as a key component of the ECM and is primarily located in vivo within joints, skin, and connective tissues.^315^ Its primary functions encompass the regulation of inflammation, vascular regeneration, and skin tissue regeneration.^316^ These functions, in turn, regulate cell proliferation, differentiation, and migration.^317^, ^318^, ^319^ Its excellent biocompatibility, biodegradability and immunogenicity contribute to its essential role in biomedicine. It finds extensive application in various medical fields including orthopedics, ophthalmology, rhinology, aesthetic medicine, and cancer treatment.^320^, ^321^ HA creates a conducive growth environment for in vivo cells. Moreover, hyaluronidase is abundantly found in the endometrium and peritoneal cavity, facilitating the complete degradation of HA.^322^

HA‐based hydrogels have received widespread attention in various medical applications based on these advantages. HA can inhibit inflammatory responses in vivo and promote vascular regeneration, thus becoming an essential barrier for endometrial regeneration.^323^ Clinical treatment with HA hydrogel may inhibit fibrotic tissue production and improve endometrial repair.^250^, ^324^, ^325^ However, the efficacy may not be good enough for patients with extensive wounds, and HA's possible role in endometrial repair and fibrosis may be amplified. Currently, most clinical studies generally recognize the endometrial repair effect of HA. In a study, SeiedeZahra Ghanadzadeh Tafti found that HA could better reduce the production of fibrotic tissue and improve endometrial repair than the control group.^231^ Another study found that the incidence of IUA decreased significantly after postoperative use of HA (OR 0.39, 95% CI 0.29–0.52).^326^ High molecular weight HA is often present in the ECM, while low molecular weight HA is frequently employed as an extracellular signaling molecule. Zhu et al.^327^ discussed the disparity in the effect of different molecular weights HA on patients with IUA after hysteroscopic surgery. Their findings revealed a reduced fibrosis percentage in the high molecular weight HA group and a more pronounced inhibitory effect on fibrosis markers (*p* < 0.05). A higher percentage of nascent endometrium was also found in the subsequent investigations.^327^ Meanwhile, Hooker et al.^251^ conducted a 42‐month‐long follow‐up. Women who applied HA hydrogels reported more sustained pregnancies and live births, with a trend toward a shorter time to conception.^251^

HA piggybacked on other agents/stem cells has been shown to promote endometrial regeneration and inhibit fibrosis. Liu et al. constructed a slow‐release system of HA hydrogel and MSCs. It was applied to a rat endometrial injury model. Comparative observations after 1 week revealed that the endometrial thickness and glandular density in the MSC‐Sec/HA group were superior to those in the control group. The premise is that HA hydrogels exhibit a porous and intact backbone that can facilitate the piggybacking of small molecules. Fluorescent labeling revealed that the injection of HP–MSCs–HA stimulated cell migration and facilitated superior repair of the functional and basal layers compared with the injection of HP–MSCs alone. Histological sections conducted 1 week postoperatively demonstrated that the HP–MSCs–HA group exhibited superior regeneration, characterized by an improved epithelial and glandular structure of the endometrium (thickness measured 292.3 ± 19.14 μm, approximately twice that of the ethanol group (171.3 ± 14.59 μm). In vitro studies demonstrated that HP–MSCs promoted cell migration and differentiation via the JNK/Erk1/2‐Stat3–VEGF pathway. Notably, VEGF, ki67, and Masson staining showed that HP–MSCs–HA promotes endometrial repair and inhibits fibrosis with a higher embryo implantation rate.^262^ The same results were obtained using HA piggyback cells.^266^

**FIGURE 4:**
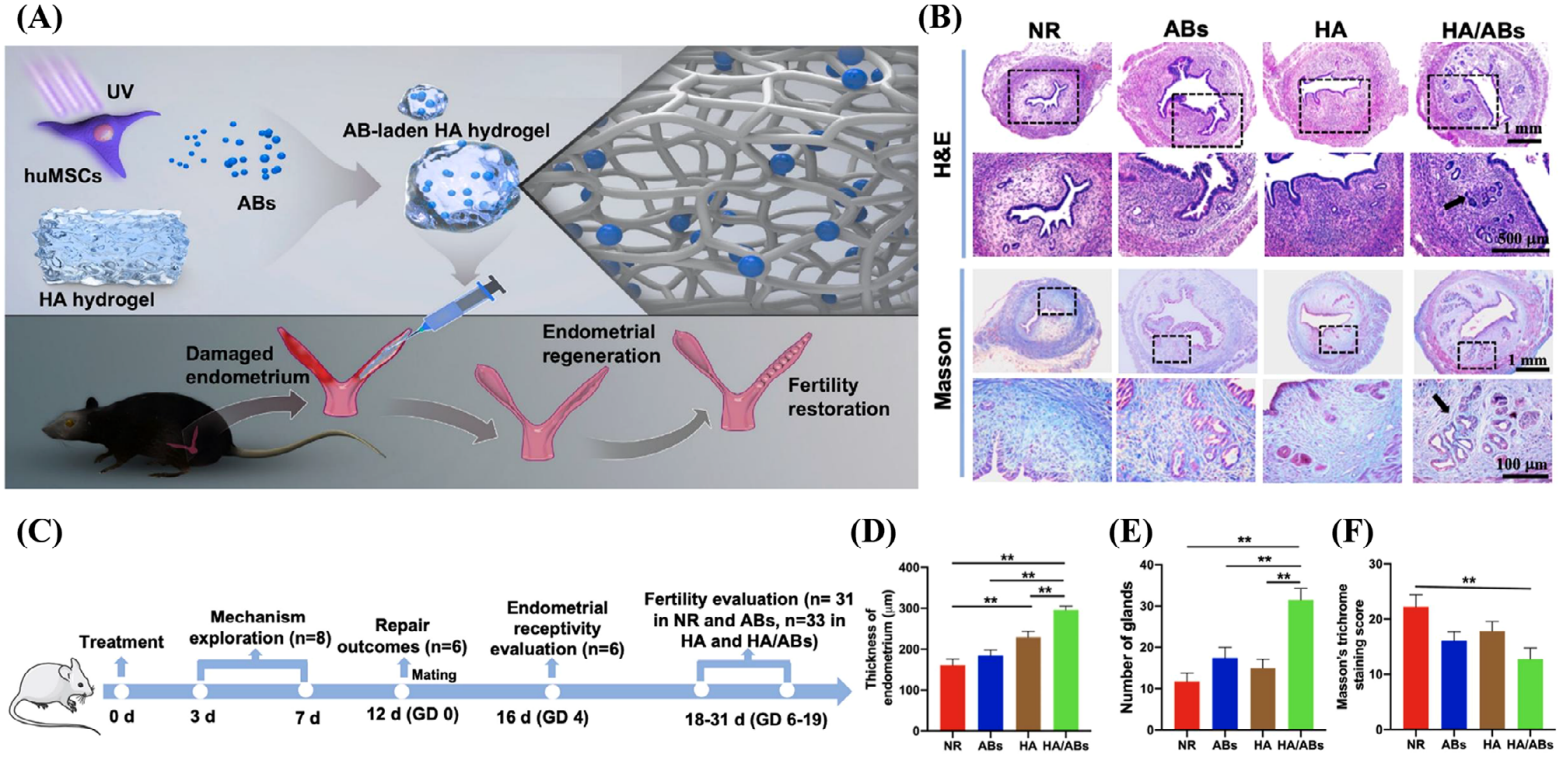
Experimental protocols for preparing and testing HA/Abs hydrogels (A and C), a hydrogel system that expresses multiple therapeutic effects and effectively promotes endometrial regeneration and vascular regeneration to prevent adhesions from occurring. (B) Staining treatment 12 d after different treatments. Black dashed boxes indicate that the following image is shown at a higher magnification. (D) (*n* = 11) and endometrial glands (E) (*n* = 11) in (B). (F) Masson's trichrome staining scores for uteri under different treatments for 12 d (*n* = 11). **p < 0.01. Reprinted with permission from Ref. 169, Copyright 2022 Bioactive Materials.

Stem cell apoptosis is often accompanied by the release of apoptotic bodies (Abs). Abs have been associated with tissue regeneration and immune regulation.^328^, ^329^ Within the field of regenerative medicine, it has been established that ABs can enhance both bone repair and skin healing.^330^, ^331^, ^332^ Xin et al.^261^ prepared Abs‐rich HA to promote endometrial repair and improve fertility outcomes. Incorporation of Abs into HA can prolong the release of Abs. HA/ABs induce macrophage immunomodulation, cell proliferation and angiogenesis in vitro. Implantation of HA/Abs increased endometrial thickness and glandular density, reduced fibrosis, and restored the normal uterine morphology, thereby promoting fertility recovery in a rat model of intractable endometrial injury. HA/Abs can enhance immunomodulatory effects. It was mainly reflected by upregulation of IL‐10 and VEGF levels and inhibition of TNF‐ɑ and TGF‐β1 expression. The final manifestation was the promotion of cell proliferation and vascular regeneration. In conclusion, the in situ administration of HA/ABs hydrogels effectively promoted endometrial regeneration and prevented re‐adhesion (Figure 4).^261^ Related studies have also confirmed the proendometrial repair and regeneration effect in large animal models.^259^, ^263^

The exceptional degradability, porosity, and mobility of HA create an optimal environment for supporting cellular activities, facilitating ideal endometrial regeneration and repair. Consequently, HA plays a crucial role in the realm of regenerative medicine by fostering endometrial repair and regeneration.

#### 4.1.3 Poloxamers

Poloxamer, also known as pluronic, is a tri‐block copolymer comprised of a hydrophilic block, polyethylene oxide (PEO), and a hydrophobic block, polyphenylene oxide. Owing to its remarkable water solubility, nontoxic properties, and sensitivity to heat, poloxamer has obtained United States Food and Drug Administration approval and has been incorporated into the U.S. and European pharmacopoeias.^333^, ^334^, ^335^ Extensive research has demonstrated that combining poloxamer with various natural or synthetic polymers or introducing additional minerals or activators makes it possible to modulate its durability, degradability, biocompatibility, and critical gelation temperature.^336^, ^337^ Consequently, this enables achieving the sol–gel transition at body temperature to the fullest extent possible. Its hydrophilic and lipophilic properties, combined with its capacity for self‐organizing micelles, render it suitable for drug delivery.^338^ Additionally, it finds extensive application in piggyback cells,^339^ proteins,^340^ growth factors,^341^ and so on. Poloxamer 188, poloxamer 127, poloxamer 237, and poloxamer 407 are frequently used in biomedical engineering.^334^, ^342^, ^343^, ^344^, ^345^ Leveraging these attributes, along with controlled gelation time and temperature, poloxamer unquestionably proves to be a valuable option for endometrial repair and regeneration. It can gel at physiological temperatures upon injection into the uterus and adapt to the diverse uterine morphologies, providing essential support.

Estrogen has been suggested to promote endometrial repair and inhibit fibrosis after hysteroscopic surgery.^346^, ^347^ Yao et al. designed a nanocomposite aloe/poloxamer hydrogel (E2@uECMNPs/AP) to carry β‐estradiol (E2) based on the theory that the combination of aloe and poloxamer has better biocompatibility.^348^, ^349^, ^350^ Nanoparticulate decellularized uterus (uECMNPs) was used to piggyback E2 (E2@uECMNPs), thus achieving delayed E2 release and increased solubility. The different constituents of E2@uECMNPs suppress the expression of TGF‐β1 and TNF‐α, while simultaneously increasing the levels of Ki67, cytokeratin, and estrogen receptor β. Specifically, these constituents stimulate stromal cell proliferation and inhibit apoptosis. The final results showed that the fibrosis and intima‐media thickness area were the most beneficial in the E2@uECMNPs/AP group.^267^ As the research continued, it was found that adding heparin had the same effect as the former, enhancing the affinity of the hydrogel system for small molecules and prolonging the half‐life in vivo.^351^ The carrier transport system (E2–HP hydrogel) was obtained by preparing heparin‐poloxamer hydrogel (HP hydrogel) loaded with E2. A study revealed that all drugs were released rapidly through dissolution when placed in a Petri dish. In contrast, the E2 release time from the HP hydrogel was significantly prolonged (more than 72 h). In contrast, E2–HP hydrogel was found to promote an increase in glandular number and inhibit fibrosis by activating the PI3K/Akt and ERK1/2 pathways.^268^ Similarly, Zhang et al.^272^ found that E2–HP hydrogel can activate the ERK1/2 and MAPKs p38 pathways and upregulate kisspeptin to promote endometrial repair and regeneration.

Hydrogel‐loaded growth factors can solve the problems of short residence time in vivo and low absorption rate in vivo.^352^, ^353^, ^354^ He et al. added ε‐polylysine (EPL), a functional excipient based on heparin–poloxamer hydrogel, to prepare endometrium‐promoting repair hydrogel (EPL–HP hydrogel). In the preparation of hydrogel, the content of EPL was changed to control the hydrogel adhesion. When keratinocyte growth factor (KGF) was encapsulated in EPL–HP hydrogel, it was found that the continuous release of the former could increase the absorption rate of KGF by trauma. It was found that the intimal thickness, the number of glands and the number of angiogenesis in the KGF–EPL–HP hydrogel group were better than those in the KGF–HP hydrogel group.^269^ He et al. also used KGF in another study but removed the function of EPL. Through ultrasound imaging and fertility evaluation, it was found that the uterine morphology and function of rats treated with KGF–HP hydrogel had the best recovery (more decadent blood vessels and higher gland density).^271^ Several reports have demonstrated the ability of KGF to promote stromal cell proliferation and inhibit apoptosis, although its reliability requires further verification.^355^, ^356^, ^357^

Hydrogels exhibit remarkable encapsulation capacity, enabling the inclusion of multiple substances. Vitamin C can impact stem cell proliferation and differentiation, mitigate inflammation, and stabilize membrane structure.^358^, ^359^ Yang et al.^270^ used PF‐127 to encapsulate bone marrow stromal cells (BMSCs) while mixing with Vitamin C. Vitamin C downregulated TNF‐a and IL‐6 levels in this process and maintained intracellular reactive oxygen species (ROS) levels. Cellular experiments revealed that BMSCs encapsulated by PF‐127 plus Vc could better maintain stem cell activity. The toxicity of PF‐127 counteracted each other. Ultimately, it promotes endometrial thickening and reduces the area of fibrosis.^270^ In conclusion, the modification of poloxamer hydrogels with heparin and Vitamin C enhances their therapeutic efficacy and permits the control of gelation temperature, further facilitates substance transport and fosters endometrial repair while diminishing fibrosis.

#### 4.1.4 The remaining polymer hydrogels

The researchers mixed different gel systems to achieve better endometrial repair and regeneration. Preparing hydrogels is mainly considered in regarding biocompatibility, delayed drug release, and hydrogel encapsulation capacity.^360^ Poly (p‐dioxane‐co‐l‐phenylalanine) (PDPA) has been shown to increase intracellular Ca^2+^ concentration and trigger calcium‐regulated phosphatase expression, ultimately leading to accelerated fibroblast apoptosis and inhibition of fibrosis.^361^ Wang et al.^277^ synthesized poly (ethylene glycol)‐b‐poly(l‐phenyl‐alanine) (PEBP) based on PDPA and made PEBP/poly (ethylene glycol) (PEG) hydrogels under hydrogen bonding. The in vitro test showed that the inhibition of L929 fibroblasts by PEBP/PEG hydrogels was increasingly effective with increasing hydrogel concentration and inhibition time. In the mouse model of uterine injury, the PEBP/PEG hydrogel‐treated group had significantly more endometrial thickness and more glands than the sham‐operated group (OS), as well as a higher embryo implantation rate at 14 days postoperatively (4.75 > 2.24 > 2.14%) and a better antiadhesion effect. Similarly, Huang added gelatin to poly(polyethene glycol citrate‐co‐N‐isopropy‐lacrylamide) (PPCN) to improve the biocompatibility of the complex.^362^ The obtained composite hydrogel (PPCNg) can achieve a reversible phase transition from liquid to solid at 37°C while having a superior progrowth effect on cells. Human amniotic MSCs (hAMSCs) were subsequently encapsulated in the hydrogels and used to evaluate the prorepair effect. Fluorescence imaging revealed that PPCNg combined with hAMSCs was more effective than direct intrauterine transplantation of hAMSCs. The higher quality of endometrial thickness and the number of glands in PPCNg combined with hAMSCs were most evident in histology. It also increased the expression of Ki‐67, CK7, CK19, VEGF, and ER/PR and inhibited the fibrotic process.^276^ Other biomedical materials include PEO–sodium carboxymethylcellulose gel,^363^ and poly (hydroxyethyl methacrylate) (PHEMA) hydrogel.^274^

With the continuous development of materials science and preparation technology, hydrogels in tissue engineering are gradually evolving toward stimulus‐responsive smart hydrogels. Stimulus‐responsive smart hydrogels are a class of smart polymeric materials that undergo deformation and phase change in the gel network in response to stimuli from the surrounding environment, which in turn causes swelling‐shrinkage or gel–sol transition,^364^, ^365^, ^366^ often manifested as changes in solubility and changes in drug release rate.^367^, ^368^ Typical medical stimuli include temperature,^369^, ^370^, ^371^, ^372^ pH,^373^, ^374^, ^375^ electric field,^376^, ^377^, ^378^ and light.^379^, ^380^ Hydrogels for tissue repair focus on temperature as an external stimulus. The occurrence of sol–gel transition under body temperature conditions is what we expect. The advantages of temperature‐sensitive hydrogels have been previously mentioned. Temperature‐sensitive hydrogels are often used in the medical field for cancer therapy,^381^, ^382^, ^383^ spinal cord regeneration,^384^ bone repair,^385^, ^386^ drug transport,^387^, ^388^ ophthalmology treatment^389^ and tissue antiadhesion.^390^ Yang et al.^391^ prepared a biodegradable and thermosensitive poly (ethylene glycol)–poly(ε‐caprolactone)–poly (ethylene glycol) (PEG–PCL–PEG; PECE) hydrogel and applied it in mouse and rat laparotomy models. PECE gels within 20 s at 37°C and adheres readily to the wound surface. In vivo, studies have shown that PECE disappears entirely in 14 days during wound healing. This protissue repair property has also been validated in animal model.^391^ Gong et al.^392^ used PECE with temperature‐sensitive properties to piggyback doxorubicin to evaluate the abdominal tissue repair performance of postoperative on this basis. Doxorubicin‐loaded PECE was found to significantly inhibit the fibrosis process, promote cell regeneration and repair and significantly reduce adhesion scores (*p* < 0.01).^392^ This study confirmed PECE temperature‐sensitive hydrogels' tissue regeneration ability and drug‐loading capacity. All related studies have affirmed the excellent repair effect of the temperature‐sensitive on abdominal tissue.^393^, ^394^

Hydrogels have exhibited promising outcomes in the domain of endometrial repair, primarily attributable to their exceptional encapsulation capacity, which aligns with the requirements of diverse drug delivery systems. Nonetheless, the majority of hydrogels can only adapt to a single intrauterine environment. In future research, it is imperative to engineer hydrogels with versatile, responsive characteristics capable of adjusting to the dynamic intrauterine environment, notably the development of multienvironment responsive hydrogels, which presents a substantial challenge requiring attention. Our objective is to facilitate drug release while effectively advancing endometrial repair and regeneration. Extensive in vitro and in vivo testing is essential to confirm hydrogel safety of hydrogels prior to large‐scale clinical trials. Nevertheless, with heightened research endeavors and interdisciplinary cooperation, we expect to significantly advance the use of hydrogels in repair and regeneration.

### 4.2 Biomedical scaffolds in cyclical endometrial repair and regeneration

Initial scaffolds employed for endometrial repair and fibrosis inhibition primarily consisted of medical‐grade silicone rubber. This material is renowned for its chemical stability, resistance to heat, resistance to oxidation, and high permeability.^395^, ^396^ Earlier experiments have confirmed the biocompatibility and absence of cytotoxicity associated with medical‐grade silicone rubber.^397^, ^398^ Nonetheless, drawbacks have arisen, including hydrophobic surfaces, limited self‐imaging compatibility, and calcification following long‐term implantation. Consequently, pursuing endometrial repair and regeneration scaffolds exhibiting outstanding performance remains a significant challenge. Porous scaffolds can function as carriers for drugs, cells, and growth factors.^399^ While hydrogels can be classified as scaffold materials, we focus more on nonhydrogel scaffolds with three‐dimensional porous structures in this section. Standard methods of scaffold preparation comprise freeze‐drying,^400^ electrospinning, microfluidics, and 3D printing.^401^ The ideal scaffold should be designed regarding biocompatibility, degradability, absorption, mechanical properties, porosity, and transport capacity. Furthermore, ensuring the scaffold's ease of use and noninflammatory nature is crucial.

#### 4.2.1 Collagen scaffolds in cyclical endometrial repair and regeneration

Collagen, the most abundant protein in mammals, accounts for 25−30% of total proteins and is widely distributed in many body tissues. As a major component of the ECM, collagen exhibits good biocompatibility, degradability, and low antigenicity, making it a popular choice in tissue engineering for applications such as wound healing, tissue repair, and drug delivery.^402^, ^403^ Porous collagen scaffolds facilitate cell adhesion and regulate cell behavior.^404^ Research has indicated that collagen scaffolds with micron‐sized pores can effectively promote cell proliferation and adhesion, with typical cells encompassing osteoblasts, chondrocytes, and keratin‐forming cells.^404^, ^405^, ^406^ Growth factors, including bFGF and VEGF, play critical roles in endometrial repair and regeneration. However, endogenous levels of growth factors are often inadequate and exogenous supplementation must be considered. Collagen scaffolds can help alleviate this deficiency while promoting vascular regeneration, among other benefits.

##### Collagen scaffolds loaded with therapeutic factor

Growth factor analogs, such as bFGF and VEGF, play crucial roles in wound healing, tissue regeneration, angiogenesis, and nerve regeneration.^407^, ^408^, ^409^ As previously mentioned, the short half‐life and rapid diffusion of exogenous growth factors in body fluids result in decreased utilization efficiency, often necessitating high doses or repeated applications to achieve therapeutic effects.^410^, ^411^ Capitalizing on the benefits of collagen, we have employed it to encapsulate growth factors and facilitate targeted transport to wound areas, thereby enhancing the wound‐healing process, which is closely tied to vascular and nerve regeneration.^412^ A research has demonstrated that collagen scaffolds piggybacking bFGF allow nerve cells to accumulate at the wound site, leading to nerve regeneration and functional recovery.^413^ Fujimaki et al.^414^ reported that collagen scaffolds loaded with bFGF accelerated neural repair in rats with significant peripheral nerve defects. Subsequently, Chen et al.^415^ designed a collagen‐based bFGF target delivery system to promote bladder regeneration, showing that the collagen/bFGF group exhibited reduced bladder contraction, faster scaffold degradation, improved bladder wall cell growth, and a lack of stone formation while ensuring a higher concentration of growth factors in the wound. However, there are still limitations in endometrial repair and regeneration.

Injuries to the abdominal wall usually result in intestinal obstruction, chronic pain and even infertility, with approximately 35% of open abdominal surgery patients readmitted to the hospital due to adhesions.^416^ Shi et al.^417^ utilized the collagen‐binding domain (CBD)–bFGF delivery system to reconstruct the injured abdominal wall. Seven days after surgery, the collagen/CBD–bFGF group had the best abdominal wall tissue regeneration with the least amount of fibrous tissue, which did not increase over time. It was observed that CBD–bFGF significantly promoted collagen remodeling, myofibril admixture and vascularization of the peritoneum at 30 days postoperatively (*p* < 0.05).^417^

Capitalizing on these benefits, Xin'an Li implemented the CBD–bFGF complex system to the uterine horns injury model and found that CBD–bFGF promoted its functional recovery and morphological reconstruction. The function of the delivery system in the different groups was tested 90 days postsurgery, revealing that the CBD–bFGF system could improve the regenerative capacity of endometrial cells and muscular cells, ultimately promoting vascular regeneration and enhancing pregnancy outcomes.^280^ VEGF is a specific mitogen for endothelial cells in vitro, acting as a proangiogenic growth factor that increases vascular permeability and promotes cell migration, among other functions.^418^ In another endometrial regeneration study, researchers introduced the CBD–VEGF carrier system into the uterine injury and closed the abdominal wall. In vitro experiments demonstrated that the CBD–VEGF carrier system increased the local VEGF concentration and prolonged the growth factor's biological effects. In vivo experiments revealed optimal tissue vascularization, thicker uterine wall, and more orderly arrangement of smooth muscle in the CBD–VEGF group. Ninety days postsurgery, the CBD–VEGF group (50%) exhibited a higher embryo implantation rate compared with PBS (6.3%) and NAT–VEGF groups (18.8%). Although no significant difference was found in the number of glands, the CBD–VEGF group had significantly more glands.^279^ Clinically relevant studies are summarized in Table 3. Collagen scaffolds loaded with growth factors may provide an innovative therapeutic for endometrial repair and regeneration. However, further researches are required to confirm its safety.

##### Collagen scaffolds loaded with stem cells

MSCs are a type of adult stem cells characterized by their inherent self‐replicative capability and the capacity for multidirectional differentiation. MSCs have garnered significant attention in the field of regenerative medicine owing to their multipotent differentiation capabilities, substantial expansion potential, and potential for immunomodulation.^419^, ^420^ Classification of MSCs frequently depends on their origin, which may include bone marrow‐derived, adipose‐derived, human menstrual blood‐derived, and umbilical cord‐derived MSCs.^421^, ^422^

Ding et al.^286^ investigated the impact of the collagen/bone marrow MSC (BM‐MSCs) complex in treating severe uterine injury (partial total hysterectomy) in rats. The collagen scaffold facilitated the growth and migration of BM‐MSCs without hindering the regular oxygen and nutrient supply. Three days posttransplantation, ELISA analysis demonstrated that the levels of bFGF, IGF‐1, TGFb‐1, and VEGF in the collagen/BM‐MSCs group surpassed those in the blank scaffold group (*p* < 0.05). Similarly, the collagen/BM‐MSCs group exhibited superior levels of bFGF, IGF‐1, and VEGF compared with the collagen/PBS and spontaneous regeneration groups (*p* < 0.05). This tendency persisted up to 30 days after transplantation, signifying a sustained uterine repair process. Pregnancy results at ninety days postsurgery revealed that the collagen/BM‐MSCs group (77.8%) had a notably higher success rate compared with the collagen/PBS (33.3%) and spontaneous regeneration groups (25%), while the sham group achieved 100% success.^286^ Thus, a cell‐loaded collagen scaffold may be an exceptional strategy to promote endometrial repair and regeneration.

**FIGURE 5:**
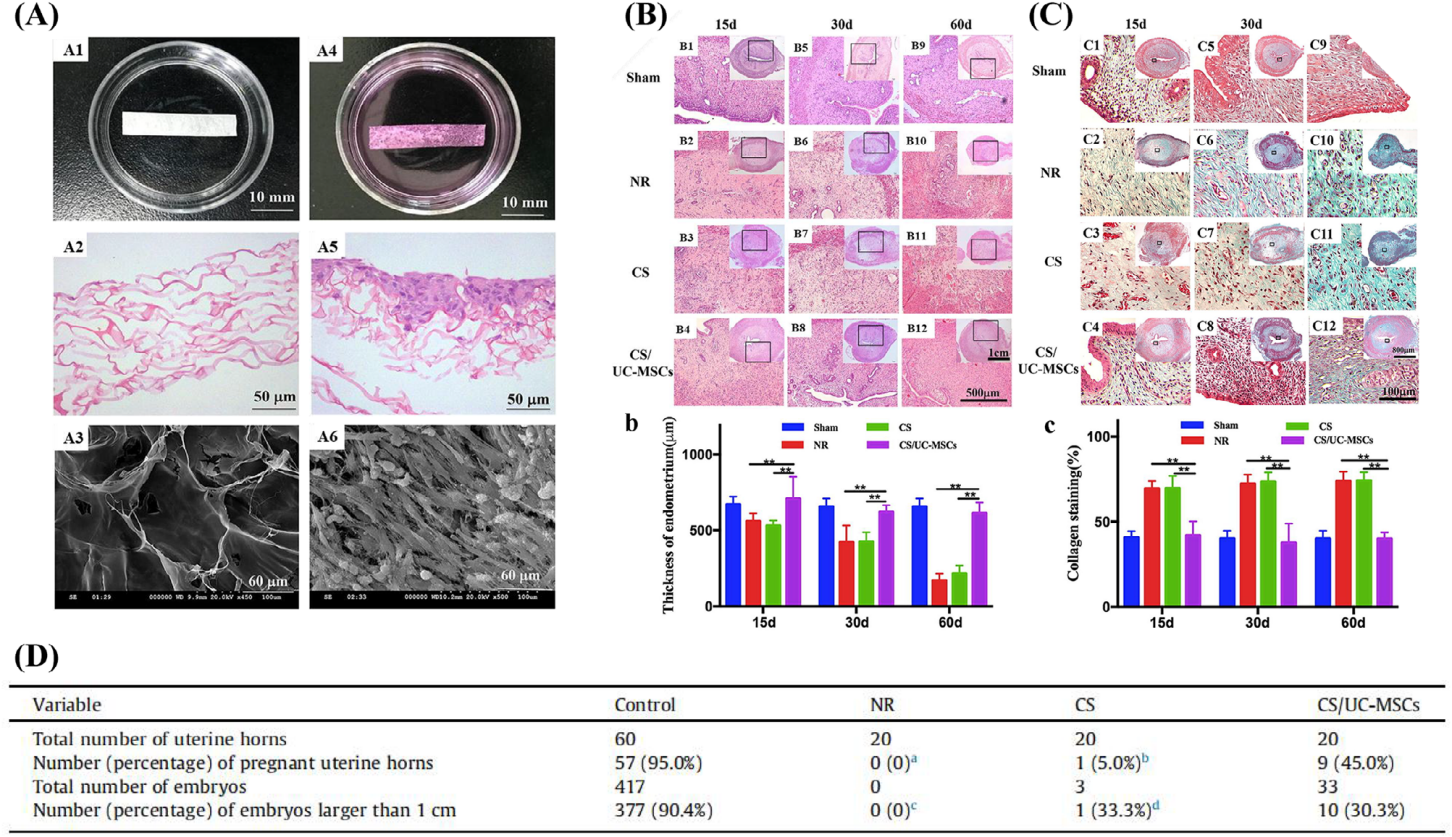
Characterization of CS/UC‐MSCs (A) and effects of different treatments on endometrial regeneration and collagen remodeling (B and C). (A) A1–A6, characterization of CS/UC‐MSCs. (A and A4) Macroscopic observation of CS (A) and CS/UC‐MSCs (A4). (A2 and A5) HE staining of CS (A2) and CS/UC‐MSCs (A5). (A3 and A6) SEM images of CS (A3) and CS/UC‐MSCs (A6). (B) HE staining of uteri after different treatments for 15 days (B1–B4), 30 days (B5–B8), and 60 days (B9–B12) in the sham group (sham) (B1, B5, and B9), the natural repair group (NR) (B2, B6, and B10), the CS group (CS) (B3, B7, and B11) and the CS/UC‐MSCs group (CS/UC‐MSCs) (B4, B8, and B12). Inserts are the corresponding overview pictures with lower magnification and the magnified regions are marked with black squares. (b) Statistical analysis of endometrial thickness after different treatments for 15 days, 30 days and 60 days. (C) Collagen staining of uteri using Masson trichrome after different treatments for 15 days (C1–C4), 30 days (C5–C8) and 60 days (C9–C12) in the sham group (sham) (C1, C5, and C9), the natural repair group (NR) (C2, C6, and C10), the CS group (CS) (C3, C7, and C11) and the CS/UC‐MSCs group (CS/UC‐MSCs) (C4, C8, and C12). Inserts are the corresponding overview pictures with lower magnification and the magnified regions are marked with black squares. (c) Statistical analysis of the percentages of collagen positive staining after different treatments for 15 days, 30 days, and 60 days. (D) Reproductive outcomes over 60 days following different treatments. (a, *p* < 0.01 NR group versus CS/UC‐MSCs group. b, *p* < 0.01 CS group versus CS/UC‐MSCs group. c, *p* < 0.01 NR group versus CS/UC‐MSCs group. d, *p* > 0.05 CS group versus CS/UC‐MSCs group). Reprinted with permission from Ref. 284, Copyright 2019 Elsevier Science & Technology Journals.

Umbilical cord MSCs (UC‐MSCs) are a promising cell therapy due to their noninvasive nature, ease of collection, low immunogenicity and excellent proliferation potential.^423^, ^424^ Xin et al.^284^ prepared porous collagen scaffolds with a pore size of 100–200 mm (0.5% w/v) by the freeze‐drying method (Figures 5A2 and A3), immersed them in 50 lL of UC‐MSCs (1 × 10^7^ cells/mL) and finally placed them into DMEM/F12‐10% FBS for 3 h to obtain CS/UC‐MSCs. The HE staining showed that the UC‐MSCs could attach well to the CS (Figure 5A5). It was observed by SEM that a large number of cells with shuttle‐like morphology were visible on the surface of CS/UC‐MSC (Figure 5A6).^284^ In an endometrial injury model, CS/UCMSCs transplantation maintained the standard luminal structure (Figures 5B4, B8, and B12). At 30 days postsurgery, the CS/UC‐MSCs group was well organized with luminal epithelium and secretory glands (Figure 5B8). By contrast, no luminal structures and disordered glands were observed in the NR group and the CS group (Figures 5B6 and B7). At 60 days posttransplantation, the endometrium recovered to normal in the CS/UC‐MSCs group (Figure 5B12). However, the NR group and the CS group exhibited severe IUAs (Figures 5B10 and B11). Endometrium thickness was greater in the CS/UC‐MSCs group than in the NR (*p* < 0.01, *n* = 6) and CS groups (*p* < 0.01, *n* = 6) (Figure 5b). Collagen remodeling of the endometrium after transplantation of CS/UC‐MSCs was assessed by Masson's trichrome staining (Figure 5C). In this experiment, we found that collagen deposition in the CS/UC‐MSCs group was similar to that of the sham‐operated group (*p* greater than 0.05, *n* = 6), and much lower than the NR group (*p* < 0.01, n = 6) and the CS group (*p* < 0.01, *n* = 6) (Figurse 5C1 and C12, c). Both in vitro and in vivo data indicated that the system enhanced cell recruitment, angiogenesis and wound healing at injured endometrial sites. Statistical embryo implantation results at 2 months revealed that CS/UCMSCs transfer prevented re‐adhesion and reestablished a fertile endometrium (Figure 5D).^284^

In another study, Xin et al.^287^ used CS to piggyback UC‐MSC‐derived exosomes (Exos), which exhibit functional similarities to UC‐MSCs but possess superior retention, stability, and ease of perfusion. The objective was to explore the effect of CS/Exos on immunomodulation during endometrial repair. Scaffold preparation was identical to previous methods, followed by the dropwise addition of exosome suspension (at a concentration of 3 × 1011/mL) to the prepared CS segment and stranding for 10 min to ensure suspension infiltration. The in vitro experiment confirmed the CS trapping function of Exos. In vivo, comparative results in mouse demonstrated that topical transplantation of CS/Exos enhanced endometrial regeneration, collagen remodeling and improved embryo implantation rates. CS/Exos increased ERα and PR in the regenerated endometrium to achieve the endometrial repair. The inflammatory response was suppressed in vitro by promoting CD163^+^ M2 macrophage polarization. In RNA‐seq, miRNAs enriched in exosomes played a significant role in inducing macrophage polarization.^287^ Liu et al.^288^ investigated the role of UC‐MSCs with degradable collagen scaffolds in rat model of endometrial injury. In the immunohistochemistry assay, CS/UC‐MSCs significantly decreased the expression of ER, CTGF, TGFβ1, and FGF2 and stimulated the expression of CK and KI67 in endometrial injury rats (*p* < 0.05). Meanwhile, CS/UC‐MSCs regulated the SDF‐1 protein level in rats with endometrial injury, ultimately inhibiting endometrial fibrosis and promoting stem cell proliferation and homing.^288^ As a result, the endometrium's ability to accept embryos was improved.

UC‐MSCs have been used for endometrial regeneration and fertility restoration in mice and have progressed to phase I clinical trials. Cao et al. designed a trial of UC‐MSCs for recurrent IUAs involving 26 patients. During standard hysteroscopic surgery, UC‐MSCs were attached to the uterine cavity lining using a Foley catheter. Postoperatively, estrogen therapy was given continuously for 30 days. Three months after surgery, all patients exhibited significant improvements in endometrial thickness and blood supply, with a mean maximum endometrial thickness increase from 4.46 ± 0.85 to 5.74 ± 1.20 mm (*p* < 0.01) and a notable reduction in adhesion scores before and after treatment. Histologically, ERα (estrogen receptor α), vimentin, Ki67, and VWF (von Willebrand factor) expression levels were upregulated relative to pretreatment levels, indicating ongoing endometrial repair and adequate vascular regeneration. At 30 months posttreatment, pregnancy outcomes significantly improved (eight patients were healthy, ten were pregnant, one was in late pregnancy, and one had an early miscarriage).^281^ Related collagen scaffold piggyback cells also include human endometrial perivascular cells (En‐PSCs),^282^ human embryonic stem cells (hESCs),^289^ and others (see Table 3 for details).

As a result of these discoveries, collagen scaffolds hold significant promise as cell carriers to support endometrial repair and regeneration. Nevertheless, challenges arise from the limitations related to implanted cell survival and uncontrolled differentiation. It is important to recognize that different collagen extraction methods can cause batch differences. Dong also proposed that the implantation of collagen scaffolds in vivo could potentially result in allergic reactions and immune rejection.^402^, ^425^, ^426^ Therefore, a substantial number of safe and reliable experiments must be undertaken to verify the appropriateness of collagen scaffolds as a therapeutic platform for endometrial repair and regeneration.

#### 4.2.2 Rest of the type of scaffolds

Gelatin methacryloyl (GelMA) is extensively used in various biomedical applications due to its excellent biocompatibility and tunable physical properties, which enable cells to proliferate and migrate in GelMA‐based scaffolds. Examples include GelMA hydrogels^427^ and GelMA scaffolds.^428^ GelMA can be a microprocessor by different methods, including 3D printing and microfluidics, to generate structures with controlled architecture.^429^ Microfluidics refers to systems that use microtubes (tens to hundreds of micrometers in size) to handle or manipulate tiny fluids (volumes ranging from nanoliters to liters). This interdisciplinary field encompasses chemistry, fluid physics, microelectronics, and biomedical engineering.^430^ Droplet microfluidics has emerged as a powerful technique for preparing submillimeter‐level cell aggregates in biomedicine for regenerative medicine and cell therapy, such as porous hydrogel microspheres and porous polymer microspheres.^431^, ^432^ Cai et al.^433^ were the first to report using GelMA/Na‐alginate (10 wt%, 2 wt%) porous scaffolds prepared by microfluidics to promote endometrial repair and regeneration. After collecting droplets (diameter of 1.2 mm and length of 1.5 cm) using hydrophobic agent‐treated capillaries, primary solidification was performed with ultraviolet (UV) light, followed by secondary solidification using 2% calcium chloride solution. The scaffold is porous, compressible, biocompatible, bio‐safe and swellable (approximately 450%), capable of rapidly recovering its original shape after deformation. It also demonstrates favorable degradability, with 40% degradation in vitro within six weeks and 30% degradation in vivo within the same timeframe. Additionally, the scaffold's excellent porous structure allows for controlled sustained release of loaded drugs/cells (by modulating droplet size to adjust pore size and transport efficiency). Animal experiments revealed that scaffolds are a physical barrier and facilitate bFGF delivery, enhancing cell recruitment, vascular regeneration and endometrial repair in the trauma area.^433^

Besides the previously mentioned scaffolds, PGS, PLGA, and ECM scaffolds find extensive application in endometrial repair and regeneration (see Table 3). Current research indicates that combining biomaterial scaffolds with drugs or cells for targeted delivery has proven effective in both human and animal models. Nonetheless, there remains a scarcity of information regarding their long‐term safety. Through the ongoing integration of tissue engineering technology, regenerative medicine, pharmacological agents, and clinical medicine, the challenges of endometrial repair and regeneration can be more effectively addressed.

### 4.3 Degradable polymeric films in cyclical endometrial repair and regeneration

Partial investigations indicate that membrane‐like barriers can promote wound repair and decrease adhesion formation by maintaining separation of the wound surface during the initial 5−7 days necessary for reepithelialization.^434^, ^435^ Several biomaterial‐based tissue‐repairing films, such as Seprafilm and Interceed,^249^, ^436^ have been developed. As an alternative to bioactive materials, barrier materials are receiving growing attention in the medical field. An ideal tissue‐repairing membrane should possess the following properties: first, excellent biocompatibility to avoid self‐inflammatory reactions.^437^ Second, biodegradability prevents the need for removal after implantation. Third, it can completely cover the wound while being convenient to use.^438^, ^439^ In 1991, O'Sullivan et al.^440^ proposed a PEG‐based peritoneal antiadhesion membrane. In recent years, membrane barriers based on PEG, PLA, and PLGA have been widely used for abdominal tissue repair.^390^, ^393^, ^394^

Azumaguchi et al.^441^ assessed the postoperative effects of silicone sheets on TCRA in a study involving 36 patients with IUAs. At the 2‐month postoperative follow‐up, it was observed that using a silicone sheet effectively prevented postoperative adhesion reconstruction and promoted endometrial regeneration. However, the disadvantages of silicone sheets have been previously mentioned. Preparing biodegradable membranes using better biocompatible, nonimmunogenic biomaterials for endometrial repair and regeneration is now widely accepted. Huberlant et al. used PEO and various amounts of d,l‐lactide (mass ratio 10:9:24) to prepare a degradable polymeric film (DPF). In a mouse model of endometrial injury, the application of DPF to cover the endometrial wound opening substantially reduced endometrial shedding and promoted endometrial formation. Only 27% of the animals in the DPF group experienced endometrium loss, whereas the HA gel and sham‐implant groups had rates of 80 and 100%, respectively. However, histological sections revealed that DPF did not alter endometrial thickness or significantly improve embryo implantation rates.^291^, ^292^ A clinical study conducted in 2022 suggests that DPF can be used clinically for endometrial injury control. The DPF used in this study was also prepared from PEO and PLA. As with its counterpart, the folded DPF unfolded rapidly within 2 h after implantation and degraded completely at a 6‐week follow‐up. Postoperative 2D ultrasound, 3D ultrasound, and hysteroscopy did not reveal adhesions or complications and the patient's signs were expected, but the endometrial thickness remained low.^291^ Related studies are shown in Table 3.

Despite the widespread use of Guardix‐sol®,^442^, ^443^ Interceed®,^444^, ^445^, ^446^ and Seprafilm®,^447^, ^448^, ^449^ and the development of numerous biomaterials to promote wound healing, there is no tissue‐repairing film that can be considered a perfect solution. The clinical application of tissue‐repairing films is often limited by a lack of awareness of the patient and social burden associated with endometrial injury and the frequent oversight of complications resulting from endometrial injury. Second, clinical evaluation of postoperative promotion of endometrial repair is challenged by the lack of clear criteria for assessing film efficacy. However, the benefits of biodegradable membranes are undeniable. Researchers should prioritize the safety of the material, followed by the possibility of combining it with anti‐inflammatory and proendometrial repair drugs to enhance the promotion of endometrial repair and regeneration. (Guardix‐sol®, Interceed®, and Seprafilm® are commonly used tissue antiadhesion products.)

### 4.4 3D‐printed scaffolds in cyclical endometrial repair and regeneration

3D printing emerged in the mid‐1990s as a material processing method for additive manufacturing and has since garnered significant attention.^450^, ^451^

In the 21st century, the development of 3D printing has revolutionized the field of regenerative medicine. For example, bioprinting involves mixing cells and proteins into printing materials and the proposed “4D printing.”^452^, ^453^ Currently, tissue engineering is increasingly embracing “3D printing,” a preparation technique that uses 3D CT to obtain the exact shape and has been used to prepare specific tissues, organs and cell cultures.^454^, ^455^, ^456^ Notably, with the increasing availability of raw materials for printing, scaffolds prepared by 3D printing may be an effective way to promote endometrial repair and regeneration.^457^

Conventional 2D cultures cannot adequately simulate endometrial tissue with 3D structures. As previously mentioned, we used hydrogels in the endometrial injury model because of their excellent 3D mesh structure properties. A study was performed to investigate the effect of polyHIPEs on endometrial cells by using emulsified templated porous polyps (known as polyHIPEs) to prepare 3D scaffolds from essential organ tissues. The results showed that polyHIPEs can promote human endometrial stromal cell adhesion, infiltration and repair functions.^458^ Nevertheless, the interchangeability of materials was one of the aspects they were looking for.

Based on the properties of hydrogels, Ji et al.^459^ prepared a hydrogel scaffold loaded with human‐induced pluripotent stem cell‐derived MSCs (hiMSC) using 3D printing. The 3D‐printing hydrogel scaffold provides a suitable growth environment without causing an autogenous inflammatory response and without cytotoxicity. One month after surgery, the 3D+hiMSC group exhibited significantly improved endometrial thickness compared with both the hiMSC group and the 3D group (360 ± 90, 210 ± 120, and 160 ± 60 μm, respectively). Subsequently, we assessed endometrial regeneration using the stained endometrial thickness‐to‐uterine wall thickness ratio and found that the 3D+hiMSC group had the best endometrial regeneration (*p* = 0.0072). Analysis of collagen deposition and gland count demonstrated that the 3D+hiMSC group had a better‐uterine morphology. Embryo implantation results at 30 days postoperatively also confirmed the superior proendometrial repair of the 3D+hiMSC scaffold.^459^ In another study, a sustained‐release microsphere (SRM) system was developed using PLGA microspheres encapsulated with granulate colony‐stimulating factor (G‐CSF), which was subsequently mixed with gelatin and alginate to obtain the 3D‐printing G‐CSF–SRM system (referred to as the 3D microsphere).^460^ The 3D microsphere was shown to promote endometrial regeneration, inhibit fibrosis, improve vascular regeneration and reconstruct normal uterine morphology in rats with endometrial injury. Subsequent embryo implantation demonstrated improved gestational function and endometrial tolerance.

The limitations of 3D printing cannot be ignored. The expensive nature of the raw materials makes it challenging to spread and specific performance requirements must be met by the printing equipment. For example, bone repair scaffolds need to meet specific mechanical properties and filling rates^460^, ^461^, ^462^; while cardiovascular scaffolds should offer an improved spatial structure and a conducive growth microenvironment for cell reproduction, facilitating cell adhesion, and growth.^463^, ^464^ These challenges can be summarized into three key points: first, maintaining cell activity and survival rate. This is followed by the aspects of cross‐linking and molding. Currently, the most commonly used is alginate solution as a raw material, which is cross‐linked and fixed after printing and then cells are inoculated onto it. The survival rate of cell inoculation is easily influenced by the cross‐linking method. The final aspect to consider is the high throughput aspect. This diversity encompasses various printing materials, including cells, proteins, and growth factors. Furthermore, the distribution of multiple materials imposes substantial demands on printing technology, and achieving seamless transitions between various printing materials remains a challenge. The development of 3D‐printing scaffold materials for endometrial repair and regeneration is promising. However, multiple challenges need to be further addressed.

## 5 STEM CELLS AND CYTOKINES IN CYCLICAL ENDOMETRIAL REPAIR AND REGENERATION

Here, we offer a comprehensive narrative review of stem cells and cytokines for endometrial repair and regeneration. The objective is to provide an in‐depth analysis that is beneficial for future research and clinical applications.

### 5.1 Advances in stem cells for cyclical endometrial repair and regeneration

Stem cells possess the ability to self‐replicate and differentiate into various cell types. Distinct from other cells, they can renew and regenerate themselves over time, making them essential for endometrial repair.^465^, ^466^ Stem cells are typically categorized into two types: embryonic stem cells and adult stem cells. Embryonic stem cells exhibit high stemness, rapid proliferation, and exceptional self‐renewal capabilities. However, their clinical utilization is restricted by ethical concerns, high tumorigenicity, and immune rejection.^70^ In contrast, adult stem cells are pluripotent cells derived from mature tissues, possessing multidirectional differentiation potential, low immunogenicity, and extensive application possibilities.^467^ The advantages of stem cells have been previously discussed and will not be reiterated here. The primary stem cell types employed for promoting endometrial repair and regeneration are as follows.

#### 5.1.1 Bone marrow MSC

BMSC is a cell category frequently employed in regenerative medicine. Initially identified in the bone marrow is a spindle‐shaped nonhematopoietic stem cell derived from the mesoderm with a strong differentiation capacity.^468^ They can differentiate into osteoblasts, chondrocytes, and hematopoietic support stroma.^469^ In 2004, Taylor et al.^470^ discovered a potential link between endometrial cells and bone marrow origin, suggesting that BMSC may be essential for endometrial repair. As we mentioned previously, a rat endometrial injury model demonstrated superior endometrial thickness and the number of glands in the transplanted group. In addition, it was found to promote the secretion of IL‐10 and reduce the levels of IL‐6 and TNF‐α.^270^, ^286^, ^471^ Recent clinical research reported that autologous BMSC transplantation led to substantial increases in both endometrial thickness and the number of glands or vessels, and some patients were able to have normal pregnancies.^472^

#### 5.1.2 Umbilical cord MSC

UCMSC is an immature cell in neonatal cord blood and cord tissue. They possess the capacity for self‐renewal and can undergo multidirectional differentiation, giving rise to various cell lineages, including neuronal cells, cardiomyocytes, and osteocytes.^473^ UCMSCs exhibit low immunogenicity, primarily characterized by downregulated expression of MHC class I molecules and the absence of MHC class II molecules and co‐stimulatory molecules (CD40, CD80, and CD86). This immunological profile facilitates allogeneic transplantation.^474^, ^475^, ^476^ Previously, it was discussed that Xin et al.^284^ employed CS/UCMSCs to facilitate endometrial repair and restore typical uterine structures. The main manifestations were induction of endometrial cell proliferation and epithelial restoration and upregulation of estrogen receptor and progesterone receptor expression. In a noncontrolled phase I clinical trial conducted by Cao et al.,^281^ collagen scaffolds loaded with UCMSCs were utilized in 30 patients with recurrent IUA‐induced infertility after surgery. We mentioned this study previously and found that patients had increased mean endometrial thickness, decreased adhesion scores, and upregulated expression levels of ERα, vimentin, Ki67, and vWF, significantly promoting endometrial repair and vascular regeneration.

#### 5.1.3 Human amniotic mesenchymal stromal cells

The amniotic membrane primarily consists of human amniotic epithelial cells (hAECs) and hAMSC. Notably, hAMSCs possess the capacity for multidirectional differentiation into various cell types, including adipocytes, hepatocytes, and neuronal cells. Additionally, the amniotic membrane offers several advantages, including abundant sources, convenient extraction, absence of ethical concerns, low immunogenicity, and nontumorigenicity.^477^, ^478^ In addition to their ability to decrease proinflammatory cytokines and increase anti‐inflammatory cytokines, as mentioned earlier, hAMSCs can also promote endometrial regeneration and repair. Li et al.^479^ performed local injections of hAMSCs into the uterine cavity to repair the endometrium. The results indicated that hAMSC significantly improved uterine structure and was accompanied by endometrial thickening, increased endometrial glands, reduced fibrosis, and increased micro angiogenesis. Importantly, the expression levels of VEGF, PCNA, and ER were increased, and finally, the pregnancy rate and the number of fetuses were increased in the treated rat.^479^

Apart from the aforementioned three commonly employed stem cells, additional stem cell types that possess the ability to facilitate molecular repair encompass human decidual MSCs (hDMSCs), menstrual blood‐derived stem cells (MenSCs), and adipose‐derived MSCs. These stem cells possess intrinsic regulatory capabilities that include proliferation and differentiation, paracrine signaling, and the ability to inhibit fibrosis and promote angiogenesis. Although the clinical application of stem cells for endometrial repair is currently in the experimental stage, numerous studies have demonstrated their remarkable efficacy and safety. In the future, stem cell technology is expected to emerge as a pivotal tool for endometrial repair, offering new prospects for patients dealing with infertility and other diseases.

### 5.2 Advances in cytokines for cyclical endometrial repair and regeneration

Currently, the primary growth factors of interest in endometrial repair are VEGF, essential fibroblast growth factor (bFGF), and KGF. Although these growth factors promote angiogenesis through different mechanisms, they are all regulated by estrogen and progesterone and act synergistically in endometrial angiogenesis and repair.

#### 5.2.1 Vascular endothelial growth factor

VEGF is a dimeric glycoprotein that functions as a peptide growth factor and acts as a specific mitogen for endothelial cells.^480^ Its primary functions include promoting heightened vascular permeability, degeneration of the ECM, migration of vascular endothelial cells, proliferation, and angiogenesis.^481^ Through long‐term observation of menstrual cycle changes, Wei et al.^482^ discovered that VEGF, bFGF, and their receptors are vital for epithelial and stromal development, as well as angiogenesis and vascular function in the endometrium of rhesus monkeys during the menstrual cycle and early pregnancy. Based on this study, Lin N promoted endometrial repair by VEGF–CBD, a piggyback system, as reflected by newborn tissues with good neovascularization, thicker endometrium and complete smooth muscle.^279^ These findings suggest that VEGF––CBD plays a crucial role in reconstructing normal uterine morphology and can repair scarring.

#### 5.2.2 Basic fibroblast growth factor

bFGF, a homolog of FGF, has an alkaline isoelectric point, is therefore called basic fibroblast growth factor.^483^ It has a solid angiogenic effect, reflected in the stimulation of cell proliferation and migration, as well as the activation of plasmin and collagenase.^408^ Clinical trials have utilized ultrasound‐mediated release of collagen bound to bFGF to treat endometrial injuries. After treatment, patients exhibited improvements in menstruation, endometrial thickness, the number of glands, and, most importantly, decreased scar area.^278^ Since we have previously discussed the relevant animal studies in the preceding article, we will not reiterate them here. Regarding bFGF, it provides a diverse effect on endometrial repair, while also promoting the invasion of trophoblast cells into the endometrium and ensuring the proper functioning of the placental vasculature.^484^ The above provides us with an excellent measure to treat endometrial injury.

#### 5.2.3 Keratinocyte growth factor

KGF, also known as fibroblast growth factor‐7 (FGF‐7), shares homology with fibroblast growth factor. KGF has been found to bind to FGF receptor 2, facilitating DNA repair in injured cells and promoting the proliferation and differentiation of epithelial cells,^485^ which is observed in the endometrium, contributing to endometrial regeneration. Due to its specific structure, it often binds to the slow‐release substance heparin. As previously mentioned, both EPL–HP hydrogel and KGF–HP hydrogel have demonstrated outstanding efficacy in endometrial repair. It is desirable to combine it with temperature‐sensitive hydrogel for endometrial repair to prevent IUAs. This combination can enhance the repair process while ensuring an appropriate release rate. The composite temperature‐sensitive hydrogel could be an essential step in restoring the standard shape of the uterus in the future.

In addition to the types mentioned above of factors, those associated with endometrial repair include stromal cell‐derived factor‐1 (SDF‐1 or CXCL12),^486^ recombinant human stomal cell‐derived factor‐1 alpha (rhSDF‐1a),^487^ and others. These growth factors not only enhance the proliferation and differentiation of endometrial cells but also facilitate the adhesion of endometrial cells, increase blood vessel formation, and thus promote endometrial recovery. However, the application of growth factors in endometrial repair is confronted with particular challenges. Some growth factors may exhibit toxic effects, and their prolonged use can lead to abnormal cell proliferation. meanwhile, growth factors' production and use costs are also high, limiting their promotion in clinical applications.

In conclusion, growth factors play a pivotal role in promoting endometrial repair and deserve increased attention and further exploration in studies to effectively address the challenges encountered in endometrial repair.

## 6 CONCLUSION AND FUTURE PERSPECTIVES

The human endometrium, the inner lining of the uterus, plays a pivotal role in the female reproductive system. It undergoes cyclic regeneration and repair during the menstrual cycle, preparing a nurturing environment for embryo implantation and supporting pregnancy. The endometrium undergoes dynamic changes during each menstrual cycle, involving the shedding of the superficial layer (menstruation) and subsequent regeneration. A delicate interplay of hormones, growth factors, and cellular interactions orchestrates this cyclic process. The endometrial repair and regeneration process can be compromised when disturbances occur, such as those resulting from conditions like EMS, fibroids, or polyps or surgical procedures like dilation and curettage (D&C). This can lead to infertility, recurrent miscarriages, or abnormal uterine bleeding.

Established clinical approaches encompass intrauterine IUD placement, intrauterine IBS placement, intrauterine HA injection, and postoperative pharmacological adjuvant therapy (e.g., estrogen, aspirin, and sildenafil) to promote endometrial repair and regeneration. Adjuvant stem cell therapy, TGF‐β inhibitor therapy inhibitor therapy, and G‐CSF therapy are alternative strategies. Nevertheless, these treatments present significant limitations in addressing endometrial injury. For instance, the cross‐linked HA employed after comprehensive hysteroscopic interventions exhibit a short residence time in the uterine cavity. Similarly, stem cell therapy has a low survival rate and faces differentiation induction and tumor formation challenges.

In recent years, regenerative medicine has emerged as a promising field for addressing endometrial repair and regeneration challenges, offering innovative solutions to restore uterine health and fertility. One of the key strategies in regenerative medicine is the use of biomaterial scaffolds. These scaffolds serve as a supportive framework for cells, promoting their attachment, proliferation, and differentiation. They possess properties like biocompatibility, biodegradability, and the ability to encapsulate bioactive substances, making them ideal candidates for endometrial repair and regeneration.

At present, research on biological materials for cyclical endometrial repair and regeneration treatment primarily focuses on: (1) animal‐ and human‐derived materials such as amniotic membrane, UBM, PRP, SIS, and so on; (2) natural polymeric materials, exemplified by HA and collagen; (3) synthetic polymeric materials, such as poloxamers, PLA‐PEO, GelMA, and so on. Standardizing performance requirements for endometrial repair and regeneration for biological scaffolds is still in progress. Based on the existing studies, these bio‐barrier materials or implanted devices should fulfill two critical criteria: adhesion prevention and endometrial repair promotion. To prevent adhesions, material properties and device structure should be designed to provide the following: (1) physical barrier: prevent adhesions between endometrium and between endometrium, material and device; (2) matching degradation periods that vary by type, as pregnancy is often achievable 3−6 months after abortion and only half a year after myomectomy; (3) degradation products that can be easily removed: the degradation products should not adhere to the endometrium and can be discharged from the cervical opening with menstrual blood or secretions to avoid adhesions or inflammation caused by the accumulation of degradation products. In promoting endometrial repair, high temperature and toxic solvents should be avoided while preparing materials or instruments to minimize posttreatment complications. Porous scaffolds or hydrogels that allow fluid exchange should be utilized to facilitate the delivery of stem cells, growth factors or drugs. By precisely regulating the release of these signaling molecules, it becomes possible to modulate cellular responses, stimulate angiogenesis (blood vessel formation), and promote a favorable environment for tissue repair. This targeted approach enhances the effectiveness of regenerative therapies while minimizing potential side effects.

Consequently, future exploration may establish and enhance standards for therapeutic scaffolds in endometrial regeneration and propose comprehensive, synergistic treatment approaches. The most promising strategies may address the challenges in cyclical endometrial repair and regeneration by integrating clinical medicine, material science, biology, and engineering technology. This process could encompass selecting suitable degradable materials, constructing scaffolds loaded with therapeutic drugs or stem cells using efficient and straightforward methods, and continuously delivering therapeutic factors precisely throughout the scaffold degradation process.

## AUTHOR CONTRIBUTIONS

*Conceived and presented the article idea and supervised the whole work*: X. H. *Collected the data and wrote the first draft of the manuscript*: H. W. *Collected the data and wrote the first draft of the manuscript*: X. Y. *Prepared the figures and tables*: Y. W. *Prepared the figures and tables*: S. Y. *Prepared the figures and participated in article revision*: D. F. *Participated in article revision*: Y. X. *Prepared the figures*: L. C. *Financial support*: K. L. *Designed the framework for the entire manuscript*: K. S. *Provided detailed guidance*: C. X. *Designed the framework for the entire manuscript, provided detailed guidance and financial support*: H. Z. *Designed the framework for the entire manuscript, provided detailed guidance and financial support*: Z. Q. All authors have read and approved the final manuscript.

## CONFLICT OF INTEREST STATEMENT

The authors declared no potential conflict of interests with respect to the research, authorship, and/or publication of this review. Author Zhiyong Qian is an Editorial board member of MedComm. Author Zhiyong Qian was not involved in the journal's review of or decisions related to this manuscript.

## ETHICS STATEMENT

Not applicable.

## Data Availability

Not applicable.
